# Inflammatory biomarkers, angiogenesis and lymphangiogenesis in epicardial adipose tissue correlate with coronary artery disease

**DOI:** 10.1038/s41598-023-30035-x

**Published:** 2023-02-17

**Authors:** Yueqiao Si, Zengbin Feng, Yixiang Liu, Wenjun Fan, Weichao Shan, Ying Zhang, Fei Shi, Enhong Xing, Lixian Sun

**Affiliations:** 1 Department of Cardiology, The Affiliated Hospital of Chengde Medical University, Chengde, 067000 Hebei China; 2 Department of Cardiology, Daping Hospital, Third Military Medical University, Chongqing, 400042 China; 3 Department of Cardiac Surgery, The Affiliated Hospital of Chengde Medical University, Chengde, 067000 Hebei China; 4 Central Laboratory of The Affiliated Hospital of Chengde Medical University, Chengde, 067000 Hebei China

**Keywords:** Cardiology, Diseases, Pathogenesis

## Abstract

In this study, we explored the relationship between inflammatory adipokine levels and coronary artery disease (CAD). We collected subcutaneous adipose tissues(SAT), pericardial adipose tissues(PAT), and epicardial adipose tissues (EAT) and serum samples from 26 inpatients with CAD undergone coronary artery bypass grafting and 20 control inpatients without CAD. Serum inflammatory adipokines were measured by ELISA. Quantitative real-time PCR and western blot were used to measure gene and protein expression. Adipocyte morphology was assessed by H&E staining. Immunohistochemistry and immunofluorescence were used to measure endothelial and inflammatory markers. Serum pro- and anti-inflammatory adipokine levels were higher and lower, respectively, in the CAD group than those in the control group (P < 0.05). In CAD, the pro-inflammatory adipokine levels via ELISA in EAT and PAT were elevated. Pro-inflammatory adipokine mRNA expression was increased, while anti-inflammatory adipokine mRNA expression decreased, in CAD relative to NCAD in EAT and PAT rather than SAT. In EAT, adipocyte area and macrophage-specific staining were lower, while lymphatic vessel marker expression was higher in CAD. Additionally, the endothelial marker expression in EAT was higher than PAT in CAD. The three tissue types had different blood vessel amounts in CAD. The regulation and imbalance expression of the novel biomarkers, including inflammatory adipokine, macrophage infiltration, angiogenesis, and lymphangiogenesis in EAT and PAT, may be related to the pathogenesis of CAD. The serum levels of inflammatory adipokines may correlate to CAD, which requires large sample size studies to get further validation before clinic practice.

## Introduction

To date, the incidence of coronary artery disease (CAD) remains high and the main cause of death worldwide^1^. However, the pathogenesis of CAD is still not clearly understood. Chronic inflammation is a key pathological mechanism of CAD that may increase aggravation of the disease^2^. Large numbers of pro- and anti-inflammatory adipokines promoting CAD development are secreted from epicardial adipose tissue (EAT)^3,4^. Previous studies have found that adipokines and inflammatory factors in EAT are associated with CAD, such as secreted frizzled-related protein 5, angiopoietin-like 4^5,6^. Complement-Clq tumor necrosis factor (TNF)-related protein (CTRP)1/9, chitinase-3-like protein 1 (YKL-40), secreted frizzled-related protein 4 (SFRP-4), salusin-β, meteorin-like (Metrnl), and salusin-α are emerging adipokines potentially correlating with CAD. EAT promotes macrophage infiltration, which secrete inflammatory factors, via directly migrating from EAT to coronary arteries during the occurrence and development of CAD^7,8^. Local inflammation and ischemia induce the production of blood vessels and lymphatic vessels, and proinflammatory factors can promote the secretion of certain vascular lymphatic modulators, such as vascular endothelial growth factors (VEGFs)^9,10^. Preclinical studies in animal models of hypercholesterolemia show that adventitial angiogenesis promotes atherosclerotic plaque progression, and that lymphatic vessels also exist and develop in parallel with blood vessels^11^. The vascular and lymphatic endothelial chemical markers CD31, CD34, podoplanin (PDPN), and lymphatic vessel endothelial hyaluronan receptor 1 (LYVE-1) are used to assess lymphatic vessel formation in tissues^12,13^. EAT, pericardial adipose tissue (PAT), subcutaneous adipose tissue (SAT) may play a different effects on myocardium due to the different biomolecules, genetic characteristics and anatomical location^14^. However, the relationship between the expression levels of the above factors in EAT, PAT, SAT and CAD remains unclear.

In this study, we investigated the expression of inflammatory adipokines and regulators of lymphatic vessel formation, as well as differences in neovascularization and lymphangiogenesis in EAT, PAT, and SAT, between CAD and non-CAD (NCAD) patients.

## Materials and methods

### Patients

In this study, 26 inpatients with angiographic CAD who underwent coronary artery bypass grafting were enrolled in the CAD group from March to September 2019, and in the same period, 20 control inpatients with chronic valvular heart disease without coronary artery stenosis needing valve replacement were enrolled in the NCAD group. The inclusion criterion for CAD patients was the presence of three-vessel disease (coronary artery stenosis ≥ 50% in the left main artery, or three branches more than 70% in any other coronary arteries). The exclusion criteria were age > 80 years, acute myocardial infarction, active chronic inflammation disease, liver or renal failure, and pharmacological glucocorticoid or immunosuppressive therapy. The baseline characteristics of the demographic and clinical information were collected by the master’s degree students. The drugs including aspirin, β-R inhibitor, statin, ACEI/ARB patients use after discharge hospitalization. Hypertension was defined as systolic blood pressure ≥ 140 mmHg and/or diastolic blood pressure ≥ 90 mmHg at rest, or previously diagnosed as hypertension in antihypertensive therapy^15^. Diabetes was defined as diabetes symptoms and random blood glucose ≥ 11.1 mmol/L, or fasting plasma glucose ≥ 7.0 mmol/L, or 2-h oral glucose tolerance test level ≥ 11.1 mmol/L, or no diabetes symptoms and at least twice blood glucose meets the above criteria^16^. Dyslipidemia was defined as serum total cholesterol ≥ 5.18 mmol/L, high-density lipoprotein cholesterol (HDL-C) ≤ 1.04 mmol/L, low-density lipoprotein cholesterol (LDL-C) ≥ 3.37 mmol/L, or triglyceride ≥ 1.7 mmol/L or previous diagnosis of dyslipidemia in medication^17^. In this study, all patients quit smoking less than 1 year. So, smoking was defined as current or prior smoking. Stroke was defined as a patient with a history of ischemic stroke.

### Blood and tissue sampling

Ten milliliters of fasting arterial blood was collected in a blood collection tube before heparinization of all patients, followed by centrifuging for 10 min at 3500 rpm, then the serum samples were collected and stored at -80 °C. EAT, PAT, SAT (500–600 mg each) were collected during the coronary artery bypass grafting or valve replacement surgery from the atrioventricular groove next to the right atrial appendage, the pericardial surface, and chest incision, respectively. Each adipose tissue sample was divided into three portions: two were frozen immediately at − 80 °C for RNA and protein extraction, the other was immersed in neutralized formalin for embedding tissue in paraffin blocks.

### Enzyme-linked immunosorbent assay (ELISA)

The serum inflammatory adipocyte factor levels were measured using an ELISA kit (Meimian Biotech Co., Ltd., Jiangsu, China) according to the manufacturer’s protocol. The pro-inflammatory adipocyte factors included tumor necrosis factor-α (TNF-α), CTRP1, YKL-40, SFRP-4, Salusin-β. The anti-inflammatory adipocyte factors included adiponectin (ADP), CTRP9, Metrnl, Salusin-α. Samples were tested in duplicate and experiments were repeated twice.

### Quantitative real-time polymerase chain reaction (RT-qPCR)

Total RNA was extracted from EAT, PAT, SAT in liquid nitrogen using TRIzol reagent (Tiangen Biotech Co., Ltd., Beijing, China) according to the manufacturer’s instructions. The concentration and purity of extracted RNA were assessed by calculating the ratio of optical density at 260 nm and 280 nm. One microgram total RNA was used as a template to be converted into cDNA using a FastQuant RT Kit (with gDNase) (Tiangen Biotech). RT-qPCR analysis was conducted using the SuperReal PreMix Plus (Tiangen Biotech) to determine the gene expression. Every mRNA amplification reaction conditions were as follows: 95 °C for 10 min, followed by 40 cycles of 95 °C for 10 s, 60 °C for 20 s, and 72 °C for 15 s. The primers for CTRP1, YKL-40, SFRP-4, CTRP9, Metrnl, vascular endothelial growth factor (VEGF)-C, VEGF-D, vascular endothelial growth factor receptor 3 (VEGFR-3), and GAPDH are shown in Table 1. GAPDH was used as the housekeeping gene, and all data were presented as relative mRNA levels. Threshold cycle values were recorded and relative gene expression was calculated using the 2 − ΔΔ CT formula (PMID: 16972087).

**Table 1:** Primers used for real-time quantitative PCR.

Gene	Forward oligonucleotides (5′–3′)	Reverse oligonucleotides(5′–3′)	Product size (bp)
CTRP1	5′-GTGCCCCAGATCAACATCAC-3′	5′-CTGCTGAGCCTGTTTTGCC-3′	281
YKL-40	5′-GACCTTGCCTGGCTCTACC-3′	5′-TGCTGTCAATGGTGACCTTC-3′	383
SFRP4	5′-CTTGCCAGTGTCCACACATC-3′	5′-TGTTCCTGCAGCCTCTCTTC-3′	346
CTRP9	5′-GCCACATTGCTGGGGTCTAT-3′	5′-AGCTTCAGCTGCAGGACAAT-3′	333
Metrnl	5′-GACCACAGGCTTCCAGTACG-3′	5′-TGGTGCAGACGGCTAGGAG-3′	311
VEGF-C	5′-AATAGTGAGGGGCTGCAGTG-3′	5′-TCGGCAGGAAGTGTGATTG-3′	419
VEGF-D	5′-TGAACGTGTTCCGATGTGGT-3′	5′-TGGCAAGCACTTACAACCTG-3′	354
VEGFR-3	5′-CCAGGATGAAGACATTTGAGG-3′	5′-AGCCGCTTTCTTGTCTATGC-3′	1298
GAPDH	5′-AGGTCATCACCATTGGCAAT-3′	5′-ACTCGTCATACTCCTGCTTG-3′	335

CTRP, complement-Clq TNF-related protein; YKL-40, Chitinase-3-like protein 1; SFRP4, secreted frizzled-related protein 4; Metrnl, Meteorin-like; VEGF, vascular endothelial growth factor; VEGFR-3, vascular endothelial growth factor receptor 3.

### Western blot

The EAT, PAT, and SAT samples were weighed and treated with Radio Immunoprecipitation Assay (RIPA) Lysis Buffer (Solarbio, Beijing, China) (300 μL/10 mg) extract protein, incubated on ice for 30 min, followed by centrifugation at 12,000 g for 10 min at 4 °C, collected the supernatants. The protein concentrations were determined using a BCA protein assay kit (Applygen, Beijing, China). Afterward, samples (50 μg of protein) were separated by 12% SDS–polyacrylamide gel electrophoresis and subsequently transferred to polyvinylidene difluoride membrane. During the test of Western blotting, the blots were cut prior to hybridization with the antibodies, according to the molecular weight, determining the position of the target protein to complete the cut of polyvinylidene difluoride membrane. Next, the samples were blocked with PBS-Tween 20 (TBST) containing 5% non-fat dry milk for 1 h at 25 °C. The membranes were incubated with primary antibody anti-CTRP9 (3:2000, Thermo Fisher Scientific, Waltham, MA, PA5-63333), anti-CTRP1 (4:1000, Thermo, PA5-20146), anti-YKL-40 (1:1000, Abcam, Cambridge, UK, ab255297), overnight at 4 °C, with anti-GAPDH used as an internal control. Followed by incubation with secondary antibodies for 1 h. The membranes were detected with chemiluminescence solution A and B mixed at a 1:1 ratio, the bands’ density was calculated by densitometric analysis using Image J 1.6.0 software.

### Hematoxylin & eosin (H&E) staining

Adipose tissue was fixed in 4% paraformaldehyde overnight, dehydrated, embedded in paraffin wax, and then cut into 5 μm-thick slices for H&E staining, immunohistochemistry, and immunofluorescence. The sections for H&E staining were subsequently deparaffined and rehydrated via a xylene and alcohol series before performing H&E staining according to standard histology procedures. The mean area of the adipocytes of each slide were measured using an automated image analysis system (ImageJ 1.6.0).

### Immunohistochemistry

The sections for immunohistochemistry were heated to 60 °C for 1 h, then dewaxed, rehydrated, and rinsed with PBS (Solarbio). Antigen retrieval was performed in sodium citrate buffer with a microwave oven for 10 min, then rinsed with PBS. Sections were incubated with primary antibodies CD68+ (Abcam), CD206+ (Santa Cruz Biotechnology, Inc., Dallas, TX, USA), CD11c+ (Abcam), PDPN (Santa Cruz Biotechnology), LYVE-1 (Abcam) overnight at 4 °C in a moist chamber, then rinsed with PBS. The sections were incubated with secondary antibodies for 30 min at 37 °C, then rinsed three times with PBS; 50–100 μL DAB reagent was added. Images were taken at a Olympas confocal microscope (Japan). The AOD of each slide was calculated from three separate fields viewed at × 400 magnification with a Nikon fluorescence microscope (Japan).

### Immunofluorescence

The sections for immunofluorescence were heated to 60 °C for 1 h, then dewaxed, rehydrated, and rinsed with PBS. Antigen retrieval was performed in sodium citrate buffer with a microwave oven for 15 min, then rinsed with PBS. Sections were incubated with primary antibodies CD31+ (Abcam), CD34+ (Santa Cruz Biotechnology), overnight at 4 °C in a moist chamber, then rinsed with PBS. Then, the sections were incubated with secondary antibodies for 1 h at room temperature, then 50–100 μL DAPI reagent was added. The images were measured using an automated image analysis system (ImageJ 1.6.0). The AOD of each slide was calculated from three separate fields viewed at × 400 magnification with a Nikon fluorescence microscope (Japan).

### Statistical analysis

Statistical analysis was performed using SPSS 19.0 (SPSS Inc., Chicago, IL, USA). All tests were two-tailed, and the differences were considered statistically significant at P < 0.05. Normality of all continuous variables were tested by Kolmogorov–Smirnov, and reported as mean value and SD or median and the interquartile range (IQR), Mann–Whitney U test was used to compare the relationship between the CAD and NCAD groups. Whereas categorical variables were expressed as percentages, the differences between the two groups were tested with chi-square test. The Kruskal–Wallis H test was used for the comparison among the three groups, and the multiple comparison between the groups was used “all pairwise”, the test level was adjusted to P = 0.0083. Correlation analysis between variables was performed using Spearman’s rank correlation coefficient. Receiver operating characteristic curve was used to determine the diagnostic efficacy and the best diagnostic cut-off point for serum inflammatory factor level.

### Ethics approval and consent to participate

This study was carried out in accordance with the World Medical Association’s Code of Ethics (Helsinki Declaration) and approved by the Institutional Review Boards of the Affiliated Hospital of Chengde Medical University (approval number LL071). Written informed consent was obtained from each patient before enrollment.

## Results

### Baseline characteristics

Patient baseline characteristics are shown in Table 2. The demographic data, including gender, age, body mass index, and smoking, were similar between the two groups (*P* > 0.05). Compared with the NCAD group, morbidity due to chest pain and hypertension were significantly higher in the CAD group (both* P* < 0.05), whereas average uric acid and blood urea nitrogen were lower in the CAD group relative to the NCAD group (all *P* < 0.05) (Table 2).

**Table 2:** Baseline characteristics of CAD and NCAD groups.

Variables	CAD group (n = 26)	NCAD group (n = 20)	χ^2^/Z	*p*
Male (%)	14 (53.8)	8 (40.0)	0.869	0.351
Age (years)	62 (51, 67)	61 (58, 63)	− 1.055	0.292
Body mass index (kg/m^2^)	25.5 ± 3.1	23.7 ± 3.8	1.929	0.062
Chest pain (%)	20 (76.9)	2 (10.0)	20.290	< 0.001
Smoking (%)	9 (34.6)	4 (20.0)	1.191	0.275
Hypertension (%)	16 (61.5)	5 (25.0)	6.038	0.014
Diabetes (%)	11 (42.3)	2 (10.0)	5.820	0.016
Dyslipidemia (%)	14 (58.3)	11 (64.7)	0.170	0.680
Ischemic stroke (%)	9 (34.6)	2 (10.0)	3.765	0.052
Uric acid (mmol/L)	297.4 ± 100.4	398.8 ± 122.1	− 2.905	0.006
Blood urea nitrogen (mmol/L)	5.40 ± 1.43	6.85 ± 1.83	− 2.789	0.008
Creatinine (μmol/L)	65.5 ± 16.3	67.6 ± 16.5	− 0.380	0.706
Aspirin (%)	24 (92.3)	1 (5.6)	204.000	< 0.001
β-R inhibitor (%)	12 (46.2)	11 (61.1)	0.545	0.329
Statin (%)	24 (92.3)	3 (16.7)	25.669	< 0.001
ACEI/ARB (%)	6 (23.1)	2 (11.1)	1.024	0.312

### Serum inflammatory adipokines levels in CAD and NCAD patients

**Figure 1:**
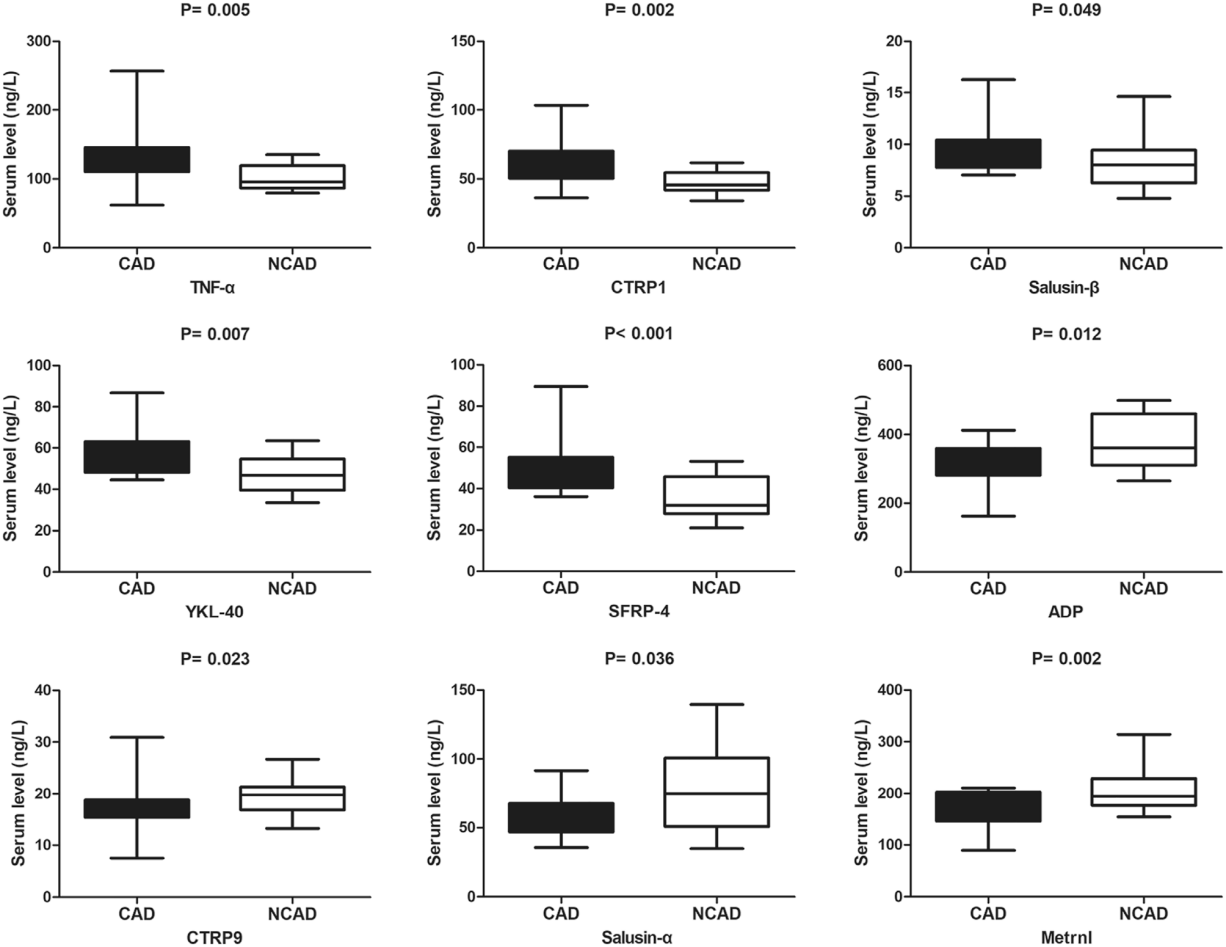
The comparison of serum inflammatory cytoadipokines levels between CAD (n = 26) and NCAD (n = 20) groups. The Mann–Whitney U test was used to compare the relationship between the CAD and NCAD groups.

Levels of the proinflammatory adipokines TNF-α, CTRP1, salusin-β, SFRP-4, and YKL-40 were significantly higher in the CAD group relative to the NCAD group (all *P* < 0.05), whereas levels of anti-inflammatory adipokines, including ADP, CTRP9, salusin-α, and Metrnl, were lower in the CAD group relative to the NCAD group (all *P* < 0.05) (Fig. 1).

### ROC curve analyses of serum inflammatory adipokines for CAD

The areas under the ROC curve (AUCs) for the proinflammatory adipokines TNF-α, CTRP1, salusin-β, YKL-40, and SFRP-4 were 0.764 [95% confidence interval (CI): 0.617–0.911; *P* = 0.006), 0.785 (95% CI: 0.643–0.927; *P* = 0.003), 0.685 (95% CI: 0.508–0.862; *P* = 0.048), 0.759 (95% CI: 0.603–0.916; *P* = 0.008), and 0.832 (95% CI: 0.699–0.965; *P* < 0.001), respectively, with the optimal diagnostic cut-off points at 99.03 ng/L, 49.06 ng/L, 7.72 ng/L, 47.71 ng/L, and 35.75 ng/L, respectively. The AUCs for the anti-inflammatory adipokines ADP, CTRP9, salusin-α, and Metrnl were 0.731 (95% CI: 0.569–0.894; *P* = 0.014), 0.708 (95% CI: 0.545–0.872; *P* = 0.023), 0.672 (95% CI: 0.492–0.851; *P* = 0.065), and 0.776 (95% CI: 0.629–0.923; *P* = 0.004), respectively, with the optimal diagnostic cut-off points at 417.4 ng/L, 18.89 ng/L, 71.76 ng/L, and 152 ng/L, respectively (Table 3).

**Table 3:** The ROC curve analysis of serum inflammatory cytoadipokines for CAD.

Variables	AUC	95%CI	*p*	Se (%)	Sp (%)	Cut off (ng/L)
TNF-α	0.764	0.617–0.911	0.006	88.0	53.3	99.03
CTRP1	0.785	0.643–0.927	0.003	83.3	66.7	49.06
Salusin-β	0.685	0.508–0.862	0.048	96.0	50.0	7.72
YKL-40	0.759	0.603–0.916	0.008	82.6	60.0	47.71
SFRP-4	0.832	0.699–0.965	< 0.001	100.0	62.5	35.75
ADP	0.731	0.569–0.894	0.014	44.4	100.0	417.4
CTRP9	0.708	0.545–0.872	0.023	68.4	77.3	18.89
Salusin-α	0.672	0.492–0.851	0.065	55.6	86.4	71.76
Metrnl	0.776	0.629–0.923	0.004	100.0	45.0	152.00

CTRP, complement-Clq TNF-related protein; YKL-40, Chitinase-3-like protein 1; SFRP4, secreted frizzled-related protein 4; ADP, adiponectin; Metrnl, Meteorin-like.

### Correlations among serum inflammatory adipokines

The proinflammatory adipokines CTRP1, salusin-β, YKL-40, and SFRP-4 were positively correlated with classic proinflammatory adipokines TNF-α (r = 0.357, 0.332, 0.383, and 0.473, respectively). CTRP1 and salusin-β were positively correlated with SFRP-4 (r = 0.509 and 0.334, respectively), and YKL-40 was positively correlated with CTRP1, salusin-β, and SFRP-4 (r = 0.710, 0.494, and 0.573, respectively). SFRP-4 was negatively correlated with ADP (r =  − 0.358). The anti-inflammatory adipokine Metrnl was positively correlated with CTRP9, salusin-α, and ADP (r = 0.377, 0.348, and 0.406, respectively) (Table 4).

**Table 4:** The correlation matrix of serum inflammatory cytoadipokines.

Variables	TNF-α	CTRP1	Salusin-β	YKL-40	SFRP-4	ADP	CTRP9	Salusin-α
CTRP1	0.357*	–	–	–	–	–	–	–
Salusin-β	0.332*	0.292	–	–	–	–	–	–
YKL-40	0.383*	0.710**	0.494**	–	–	–	–	–
SFRP-4	0.473**	0.509**	0.334*	0.573**	–	–	–	–
ADP	− 0.550**	− 0.371*	− 0.171	− 0.183	− 0.358*	–	–	–
CTRP9	− 0.094	− 0.202	− 0.121	− 0.137	− 0.064	0.237	–	–
Salusin-α	− 0.311	− 0.002	0.070	0.123	− 0.185	0.250	− 0.054	–
Metrnl	− 0.338	− 0.038	− 0.096	0.022	− 0.308	0.406*	0.377*	0.348*

CTRP, complement-Clq TNF-related protein; YKL-40, Chitinase-3-like protein 1; SFRP4, secreted frizzled-related protein 4; ADP, adiponectin; Metrnl, Meteorin-like.

*P < 0.05, **P < 0.01.

### Inflammatory adipokine expression in adipose tissues

**Figure 2:**
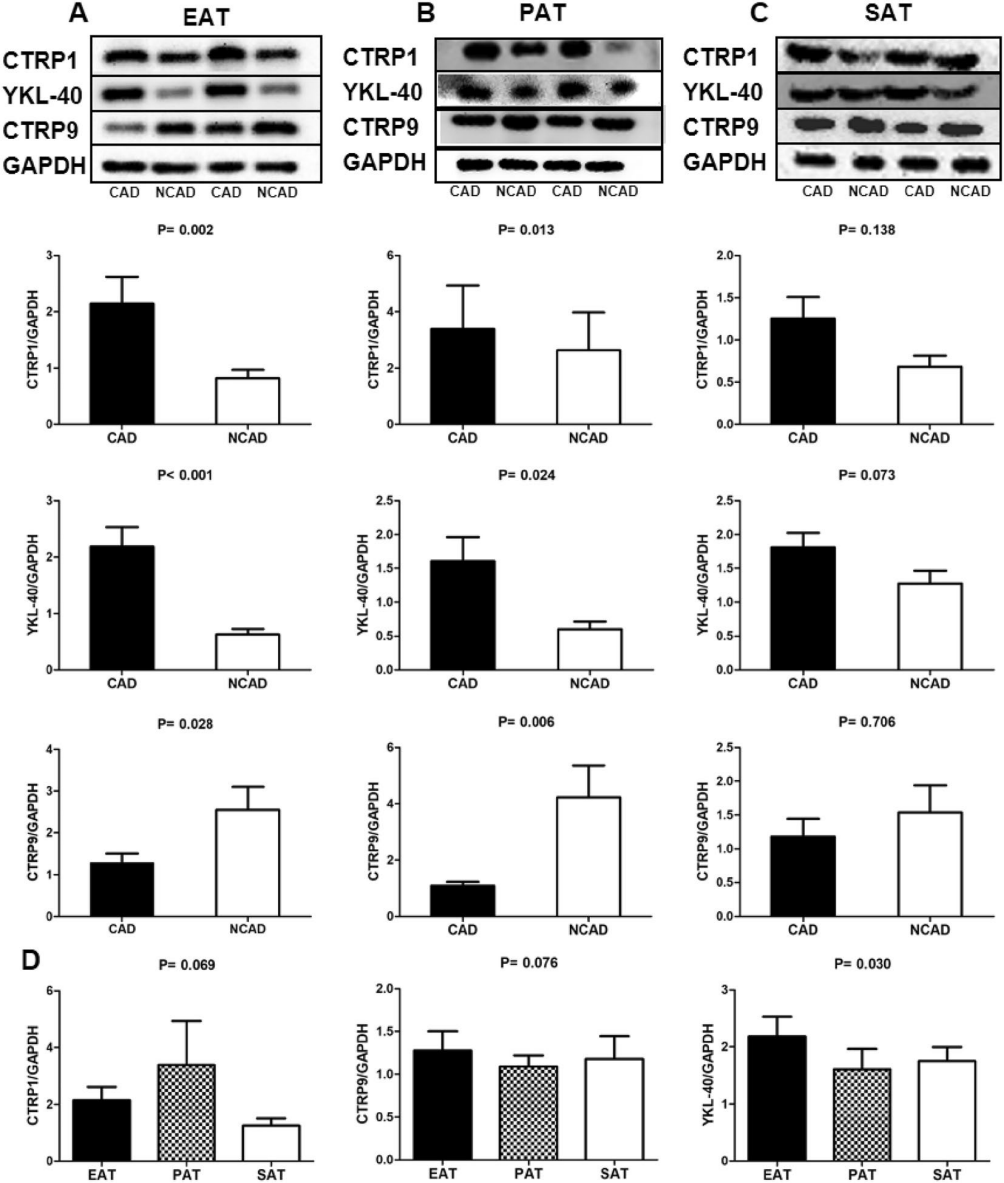
Inflammatory cytoadipokines protein expression levels in adipose tissues in CAD (n = 26)and NCAD (n = 20) groups. (**A**) Representative bands and quantitative analyses of CTRP1, YKL-40, CTRP9 protein expression in EAT. (**B**) Representative bands and quantitative analyses of CTRP1, YKL-40, CTRP9 protein expression in PAT. (**C**) Representative bands and quantitative analyses of CTRP1, YKL-40, CTRP9 protein expression in SAT. (**D**) Quantitative analyses among EAT, PAT, SAT of CTRP1, YKL-40, CTRP9 protein expression in CAD group (n = 26). The Mann–Whitney U test was used to compare the relationship between the CAD and NCAD groups. The Kruskal–Wallis H test was used for the comparison among the three groups, and the multiple comparison between the groups was used “all pairwise”, the test level was adjusted to P = 0.0083. Source data was the average relative density of immunoblot bands of all adipose tissue samples in CAD and NCAD patients. The cropped inserts show representative immunoblot examples for CTRP1, CTRP9, YKL-40, GAPDH of 3 adipose tissue. The original immunoblots are available in Supplementary Figs. S1–S12; *P* values resulting from comparison of CAD group and NCAD group means of concentration values through the Wilcoxon test are written above each boxplot pair. During the test of Western blotting, the blots were cut prior to hybridization with the antibodies.

Figure 2 shows that CTRP1 and YKL-40 levels in EAT and PAT were significantly elevated in the CAD group relative to the NCAD group (*P* < 0.05), whereas CTRP9 levels in EAT and PAT was significantly reduced in the CAD group relative to the NCAD group (*P* < 0.05). However, CTRP1, YKL-40, and CTRP9 levels in SAT were not statistically different between the CAD and NCAD groups (all *P* > 0.05) (Fig. 2). Evaluation of differences in CTRP1, YKL-40, and CTRP9 levels among EAT, PAT, and SAT revealed that only YKL-40 level differed among the three adipose tissues, with that in PAT lower than that in EAT and SAT (all *P* < 0.05) (Fig. 2).

### Adipokine mRNA levels in adipose tissues

**Figure 3:**
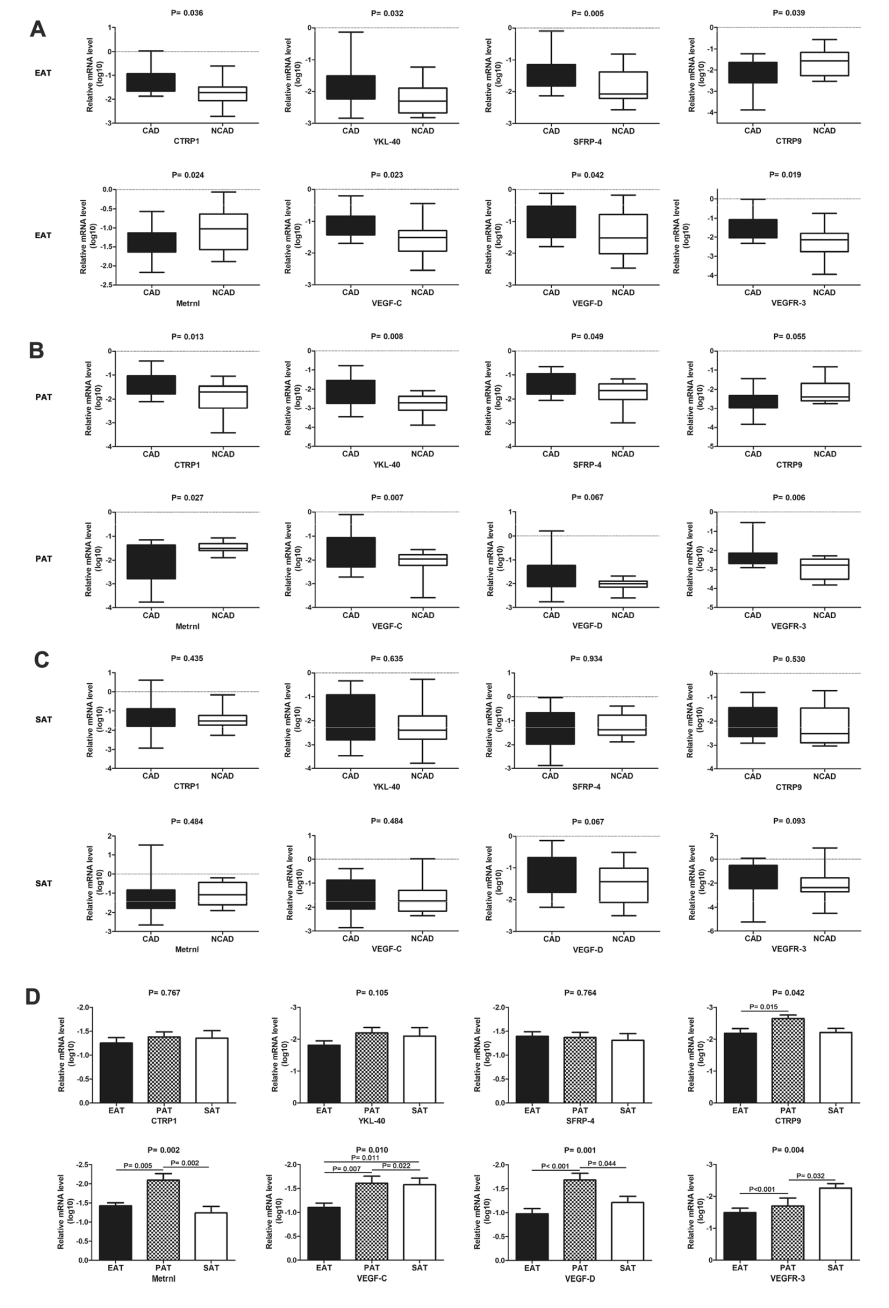
Cytoadipokines mRNA expression in adipose tissues in CAD (n = 26) and NCAD (n = 20) groups. (**A**–**C**) Quantitative analyses of CTRP1, YKL-40, SFRP-4, CTRP9, Metrnl, VEGF-C, VEGF-D, VEGFR-3 mRNA expression in EAT (**A**), PAT (**B**), SAT (**C**). (**D**) Quantitative analyses between EAT, PAT, SAT of CTRP1, YKL-40, SFRP-4, CTRP9, Metrnl, VEGF-C, VEGF-D, VEGFR-3 mRNA expression in CAD group (n = 26). The Mann–Whitney U test was used to compare the relationship between the CAD and NCAD groups. The Kruskal–Wallis H test was used for the comparison among the three groups, and the multiple comparison between the groups was used “all pairwise”, the test level was adjusted to P = 0.0083.

mRNA levels of the proinflammatory adipokines *CTRP1*, *YKL-40*, and *SFRP-4* were significantly higher in the CAD group relative to the NCAD group in EAT and PAT (all *P* < 0.05), although the difference was not statistically significant between the two groups in SAT (all *P* > 0.05). In EAT, mRNA levels of the anti-inflammatory adipokines *CTRP9* and *Metrnl* were significantly decreased in the CAD group relative to the NCAD group (both *P* > 0.05). In PAT, *CTRP9* mRNA level was significantly lower in the CAD group relative to the NCAD group (*P* < 0.05), and *Metrnl* mRNA level was not statistically different between the two groups (*P* > 0.05). Additionally, differences in *CTRP9* and *Metrnl* mRNA levels were not statistically significant in SAT between the two groups (both *P* > 0.05). Moreover, mRNA levels of *VEGF-C*, *VEGF-D*, and *VEGFR-3* were significantly higher in the CAD group relative to the NCAD group in EAT (all *P* < 0.05), and *VEGF-C* and *VEGFR-3* levels were significantly higher in the CAD group relative to the NCAD group in PAT (both *P* < 0.05). Furthermore, mRNA levels of *VEGF-C*, *VEGF-D*, and *VEGFR-3* in SAT were not statistically different between the two groups (all *P* > 0.05) (Fig. 3).

### Correlations among cytoadipokine mRNA levels in EAT and PAT

Correlations among adipocyte mRNA levels in EAT are shown in Table 5. *CTRP1*, *SFRP-4*, and *YKL-40* levels were positively correlated with *VEGF-C*, *VEGF-D*, and *VEGFR-3* levels; *CTRP9* level was positively correlated with *VEGF-C* and *VEGF-D* levels; and *Metrnl* level was positively correlated with *VEGFR-3* level (all *P* < 0.05). Additionally, *CTRP1* level was positively correlated with *SFRP-4*, *YKL-40* and *Metrnl* level; *SFRP-4* level was positively correlated with *YKL-40* level; and *CTRP9* level was positively correlated with *Metrnl* level (all *P* < 0.05). As are shown in Table 6, *CTRP1* level was positively correlated with *SFRP-4*, *VEGF-D*, and *VEGFR-3* levels; and *SFRP-4* level was positively correlated with *YKL-40* and *VEGFR-3* levels (all *P* < 0.05).

**Table 5:** The correlation matrix of cytoadipokines mRNA expression in EAT.

Variables	CTRP1	SFRP-4	YKL-40	VEGF-C	VEGF-D	VEGFR-3	CTRP9
SFRP-4	0.651**						
YKL-40	0.751**	0.706**					
VEGF-C	0.564**	0.440**	0.561**				
VEGF-D	0.573**	0.446**	0.517**	0.821**			
VEGFR-3	0.741**	0.752**	0.584**	0.369*	0.526**		
CTRP9	0.331	0.091	0.331	0.457**	0.391*	0.204	
Metrnl	0.435*	0.291	0.287	0.314	0.149	0.452**	0.519**

CTRP, complement-Clq TNF-related protein; SFRP4, secreted frizzled-related protein 4; YKL-40, Chitinase-3-like protein 1; VEGF, vascular endothelial growth factor; VEGFR-3, vascular endothelial growth factor receptor 3; Metrnl, Meteorin-like.

*P < 0.05, **P < 0.01.

**Table 6:** The correlation matrix of cytoadipokines mRNA expression in PAT.

Variables	CTRP1	SFRP-4	YKL-40	VEGF-C	VEGF-D	VEGFR-3	CTRP9
SFRP-4	0.586**						
YKL-40	0.258	0.372*					
VEGF-C	0.323	0.196	0.288				
VEGF-D	0.496**	0.362	0.303	0.112			
VEGFR-3	0.430*	0.499**	0.299	0.554**	0.215		
CTRP9	0.132	− 0.152	− 0.225	− 0.141	0.256	− 0.142	
Metrnl	0.133	0.334	− 0.217	0.229	− 0.137	0.085	0.262

CTRP, complement-Clq TNF-related protein; SFRP4, secreted frizzled-related protein 4; YKL-40, Chitinase-3-like protein 1; VEGF, vascular endothelial growth factor; VEGFR-3, vascular endothelial growth factor receptor 3; Metrnl, Meteorin-like.

*P < 0.05, **P < 0.01.

### Histology

**Figure 4:**
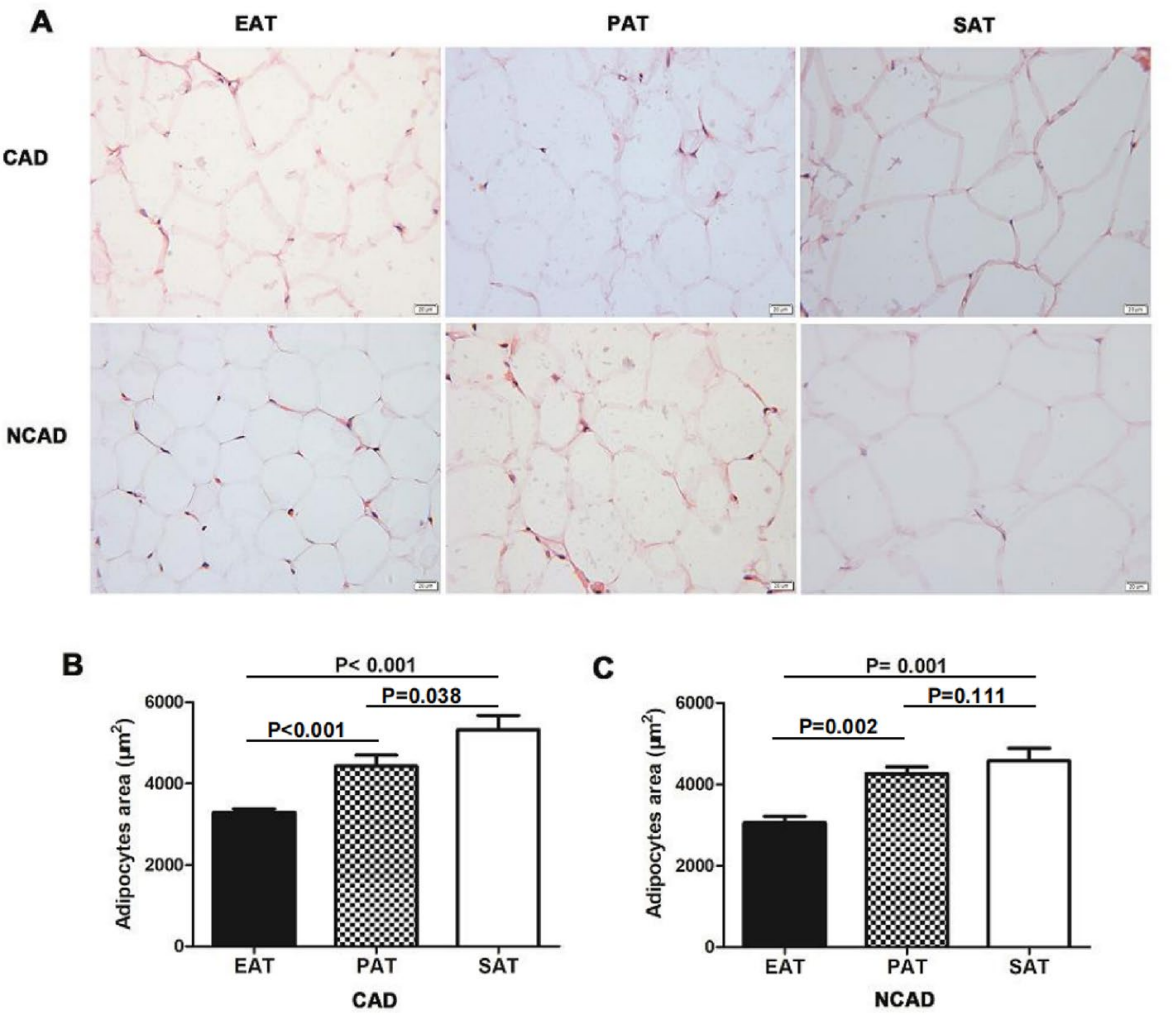
The adipocytes area of EAT, PAT and SAT. (**A**) Representative images of adipocytes area of EAT, PAT and SAT in CAD (n = 20) and NCAD group (n = 12). (**B**) Quantitative analyses of adipocytes area between EAT, PAT and SAT in CAD group (n = 20). (**C**) Quantitative analyses of adipocytes area between EAT, PAT and SAT in NCAD group (n = 12). The Mann–Whitney U test was used to compare the relationship between the CAD and NCAD groups.

The mean adipocyte areas of EAT, PAT, and SAT in CAD patients were 3241.9 μm^2^, 4180.7 μm^2^, 5545.9 μm^2^, respectively, whereas those in NCAD patients were 3046.5 μm^2^, 4115.2 μm^2^, and 4813.7 μm2, respectively. Adipocyte morphology in the three tissues differed, with the adipocyte area of EAT significantly smaller than that of PAT and SAT (both *P* < 0.05), whereas there was no statistical difference in the adipocyte areas of PAT and SAT in NCAD patients (*P* > 0.05) (Fig. 4).

### Macrophage infiltration in adipose tissues

**Figure 5:**
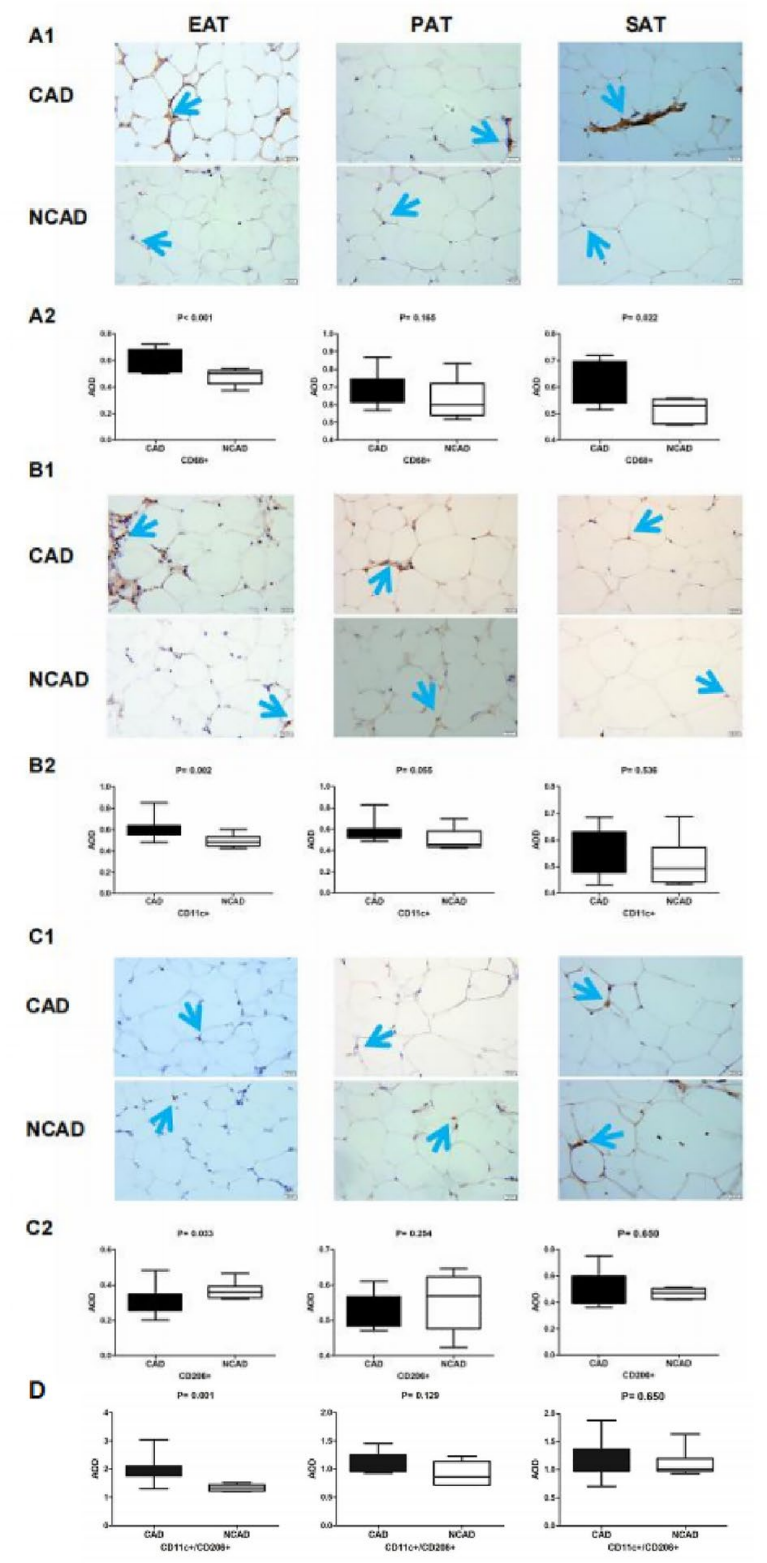
Immunohistochemistry detection of macrophage infiltration in adipose tissues in CAD (n = 11) and NCAD groups (n = 9). (**A1**) Representative images of CD68+ expression in EAT, PAT and SAT. (**A2**) Average Optical Density (AOD) of CD68+ in EAT, PAT and SAT between CAD and NCAD patients. (**B1**) Representative images of CD11c+ expression in EAT, PAT and SAT. (**B2**) AOD of CD11c+ in EAT, PAT and SAT. **C1**, Representative images of CD206+ expression in EAT, PAT and SAT. (**C2**) AOD of CD206+ in EAT, PAT and SAT. (**D**) Quantitative analyses of CD11c+/CD206+ in EAT, PAT and SAT. The Mann–Whitney U test was used to compare the relationship between the CAD and NCAD groups.

Macrophage infiltration determined by CD68 immunostaining showed significantly greater in EAT and SAT in the CAD group relative to the NCAD group (all *P* < 0.05); however, PAT macrophage infiltration was not statistically different between the CAD and NCAD groups (*P* > 0.05). We then determined differences in the M1 and M2 macrophage phenotypes by immunohistochemical staining for CD11c and CD206, respectively. We found that the number of CD11c+ macrophages was significantly increased and that of CD206+ macrophages significantly decreased in EAT from CAD patients relative to NCAD patients (both *P* < 0.05). In SAT, there was no statistical difference between CD11c+ and CD206+ macrophages between the CAD and NCAD groups (both *P* > 0.05). Additionally, calculation of the CD11c+/CD206+ ratio, which reflects macrophages shift into an inflammatory state, revealed that CD11c+/CD206+ macrophage ratio was significantly increased only in EAT in CAD patients when compared with NCAD patients (*P* < 0.05) (Fig. 5).

### Expression of the lymphatic markers PDPN and LYVE-1 in adipose tissues

**Figure 6:**
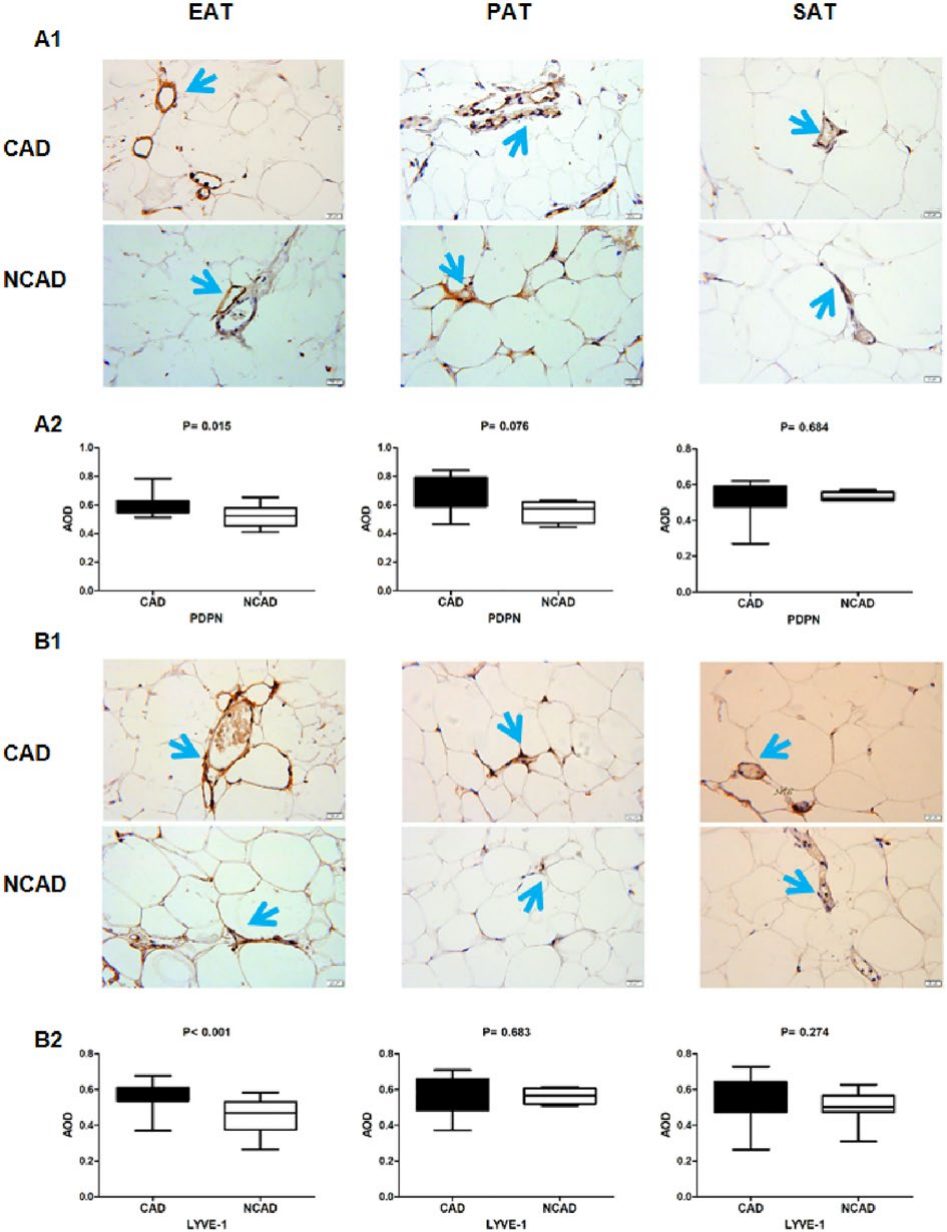
Immunohistochemistry detection of PDPN and LYVE-1 expression in adipose tissues. (**A1**) Representative images of PDPN expression in EAT, PAT and SAT. (**A2**) AOD of PDPN in EAT, PAT and SAT between CAD (n = 22) and NCAD (n = 15) patients. (**B1**) Representative images of LYVE-1 expression in EAT, PAT and SAT. (**B2**) AOD of LYVE-1 in EAT, PAT and SAT (n = 22). The Mann–Whitney U test was used to compare the relationship between the CAD and NCAD groups.

PDPN and LYVE-1 levels in EAT were significantly higher in the CAD group relative to the NCAD group (both *P* < 0.05), whereas their levels in PAT and SAT were not statistically different between the two groups (both *P* > 0.05). Additionally, PDPN level in CAD patients differed among the three tissue types, with levels in SAT lower than those in EAT and SAT (all *P* < 0.05); however, there were no significant differences in LYVE-1 levels in CAD patients among the three tissue types (*P* > 0.05) (Fig. 6).

### Expression of the vascular markers CD31 and CD34 in adipose tissues

**Figure 7:**
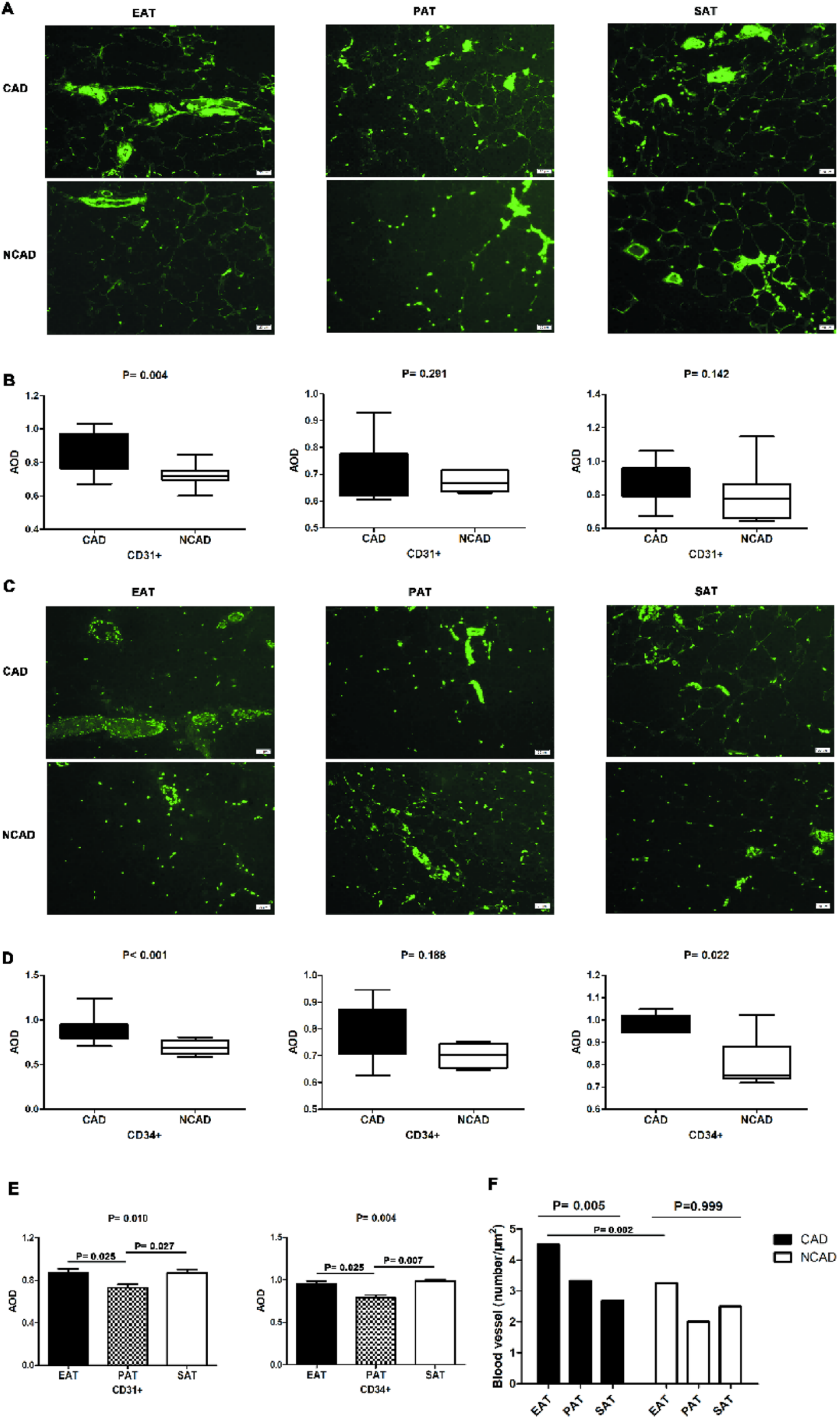
Immunofluorescence detection of CD31+ and CD34+ expression in adipose tissues in CAD (n = 19) and NCAD groups (n = 9). (**A**) Representative images of CD31+ expression in EAT, PAT and SAT. (**B**) AOD of CD31+ in EAT, PAT and SAT. **C**, Representative images of CD34+ expression in EAT, PAT and SAT. (**D)**, AOD of CD34+ in EAT, PAT and SAT. (**E**) AOD analyses of PDPN, LYVE-1 between EAT, PAT and SAT in CAD patients. (**F**) The number of blood vessels in EAT, PAT and SAT. The Mann–Whitney U test was used to compare the relationship between the CAD and NCAD groups. The Kruskal–Wallis H test was used for the comparison among the three groups, and the multiple comparison between the groups was used “all pairwise”, the test level was adjusted to P = 0.0083.

The numbers of CD31+ and CD34+ cells in EAT were significantly higher in the CAD group than in the NCAD group (both *P* < 0.05), although no statistical difference in PAT was observed between groups (both *P* > 0.05). In SAT, the number of CD34+ cells was higher in CAD patients relative to NCAD patients (*P* < 0.05), whereas the number CD31+ cells did not differ significantly between the CAD and NCAD groups (*P* > 0.05). The numbers of CD31+ and CD34+ cells in CAD patients differed among the three tissue types, with those in PAT significantly lower relative to EAT and SAT (all *P* < 0.05), although no significant difference was observed between SAT and PAT (*P* > 0.05) (Fig. 7).

The numbers of blood vessels in EAT, PAT, and SAT from CAD patients were 4.5, 3.3, and 2.7, respectively, whereas those from NCAD patients were 3.25, 2.0, and 2.5, respectively. The numbers of blood vessels in the three tissue types from CAD patients differed significantly (*P* < 0.05), whereas those from NCAD patients did not differ significantly (*P* > 0.05). The number of blood vessels in EAT from CAD patients was the largest (Fig. 7).

## Discussion

Based on findings from previous animal studies^18^, the present study investigated the relationship between neovascularization, lymphangiogenesis, and inflammatory adipocytokine levels in EAT, PAT, and SAT from human subjects. The proinflammatory adipokines CTRP1, salusin-β, YKL-40, and SFRP-4 and the anti-inflammatory adipokines CTRP9, salusin-α, and Metrnl are newly discovered inflammatory adipokines that may correlate with CAD^19–21^. The present study is the first to investigate the expression of these adipokines in adipose tissues and compare differences in their expression between EAT, PAT, and SAT. In CAD patients, mRNA and protein levels of proinflammatory adipokines were elevated in both EAT and PAT, whereas levels of anti-inflammatory adipokines were reduced relative to those in NCAD patients. However, there were no significant differences in gene and protein expression of pro- factors and anti-inflammatory factors in SAT from CAD and NCAD patients. Previous studies found that secretion of proinflammatory adipokines increased and anti-inflammatory adipokines decreased in the EAT of rodents, resulting in upregulated development of atherosclerosis^18,22^; however, there have been few studies on EAT and PAT from CAD patients. Furthermore, the roles of CTRP1, YKL-40, SFRP-4, CTRP9, and Metrnl secreted by EAT and PAT in CAD remain unclear, and research on this topic is still in the exploratory stage. The current evidence shows that levels of secreted CTRP1, YKL-40, and SFRP-4 from EAT or PAT are elevated in CAD patients, whereas those of CTRP9 and Metrnl are lower relative to NCAD patients. Variations in these molecular signatures might related to atherosclerosis.

Atherosclerosis is an imbalanced chronic inflammatory reaction in the arterial wall caused by certain stimuli and that produces a wide range of inflammatory molecules, which diffuse into the blood circulation and provide information regarding the existence and status of disease during its development^23^. In the present study, we found that serum levels of proinflammatory adipokines (TNF-α, CTRP1, salusin-β, YKL-40, and SFRP-4) were elevated in CAD patients, whereas those of anti-inflammatory adipokines (ADP, CTRP9, salusin-α, and Metrnl) were lower relative to those in NCAD patients. Intimal atherosclerosis stimulates the secretion of proinflammatory adipokines and inhibits the release of anti-inflammatory adipokines, thereby affecting cardiac metabolism and inflammation and exerting crucial proinflammatory and pro-atherogenic effects in vivo^24^. Therefore, CTRP1, salusin-β, YKL-40, SFRP-4, CTRP9, salusin-α, and Metrnl may play important roles in CAD.

Previous studies show that adipocytokines affect each other, with proinflammatory adipokines promoting the secretion of other types of proinflammatory adipokines and inhibiting anti-inflammatory adipokine expression^25^. Atherosclerosis entails a complex interaction of risk factors, some of which stimulate inflammation and promote the secretion of inflammatory adipokines^26^. In the present study, we found that mRNA and protein levels of proinflammatory factors positively correlated with other levels of proinflammatory factors, with the same correlations observed for anti-inflammatory factors. Previous studies demonstrated that inflammatory adipocytokines can be used as biomarkers of atherosclerosis^3,27^. In the present study, we showed that serum levels of CTRP1, salusin-β, YKL-40, SFRP-4, CTRP9, salusin-α, and Metrnl might represent CAD biomarkers according to ROC analysis.

Additionally, we found that CD68+ M1 macrophages and the CD11c+/CD206+ ratio were significantly elevated in EAT from CAD patients relative to NCAD patients, whereas the number of M2 macrophages was significantly reduced. Macrophages are immunocompetent cells that response to local inflammation, and adipose tissue exhibiting chronic inflammation in CAD patients harbors abundant amounts of macrophages^28^. Activated M1 and M2 macrophages perform different functions by secreting either proinflammatory or anti-inflammatory factors^29^. Moreover, M1 macrophages exert strong microbicidal activities capable of inhibiting the proliferation of surrounding cells and damaging adjacent tissue, whereas M2 macrophages play an anti-inflammatory role by removing debris and promoting the proliferation of adjacent cells and tissue repair. The M1/M2 macrophage ratio reflects a shift in the state of local inflammation^30^. In the present study, the results showed macrophages in EAT from CAD patients were predominantly of the M1 phenotype associated with CAD.

Furthermore, we found that the number of blood vessels increased in EAT from CAD patients along with a significantly higher populations of CD31+ and CD34+ cells in EAT relative to those observed in NCAD patients. CD31 and CD34 are markers of angiogenesis, with CD31 a signaling-related glycoprotein abundant on vascular endothelial cells^31^ and CD34 a transmembrane phosphoglycoprotein on the surface of vascular endothelial progenitor cells, hematopoietic stem cells, and hematopoietic progenitor cells^32^. Inflammation is an important trigger of angiogenesis in ischemic tissues and can promote the secretion of pro-angiogenic factors and the proliferation of endothelial cells, with this also pro-angiogenic effect also accelerated by some inflammatory cytokines^10^. In the CAD group, we observed increased neovascularization which may be a useful pathological founding.LYVE-1 is the main receptor of hyaluronic acid in the endothelium of lymphatic vessels^33^, and PDPN is expressed in lymphatic endothelial cells and fibroblastic reticular cells in lymph nodes and capable of promoting lymphangiogenesis and lymphatic invasion^33,34^. In the present study, we found significantly elevated PDPN and LYVE-1 levels in EAT from CAD patients relative to NCAD patients. Inflammation is aggravated and lymphatic drainage is insufficient in atherosclerotic lesions, with various stimuli promoting the generation of lymphatic vessels^35^, which often occurs in parallel to angiogenesis. Ioannis et al.^36^ demonstrated increased lymphangiogenesis in adipose tissue around atherosclerotic plaques and accompanied by higher expression of lymphangiogenesis regulators. VEGF-C, VEGF-D, and VEGFR-3 regulate vacsular growth in endothelial tissue and promote the proliferation and migration of vascular endothelial cells, thereby playing an important role in lymphangiogenesis and angiogenesis^37^. In the present study, mRNA levels of *VEGF-C, VEGF-D,* and *VEGFR-3* were higher in EAT, and VEGF-C and VEGFR-3 levels were higher in PAT from CAD patients relative to NCAD patients, with correlations also observed between VEGF-C, VEGF-D, and VEGFR-3 levels and the number of inflammatory adipocytes. These results suggest that neovascularization and lymphangiogenesis in EAT and the number of inflammatory adipokines related to CAD.

We observed differences in mRNA levels of *CTRP9, Metrnl, VEGF-C, VEGF-D,* and *VEGFR-3,* protein expression of YKL-40, and levels of CD31, CD34, PDPN, CD206 and the CD11c+/CD206+ ratio among the three adipose tissues. EAT and PAT constitute a unique paracrine visceral fat reservoir with unique anatomical, biomolecular, and genetic characteristics that regulates the production and metabolism of the adjacent myocardium^14,38^. However, the embryology and blood supply of EAT and PAT differ, which were observed in their metabolic and physiologic properties^39^. In the present study, the adipocyte areas were found significantly smaller in EAT relative to PAT and SAT in CAD patients and NCAD patients. Although EAT, PAT, and SAT constitute white adipose tissues, in addition to the different anatomical positions of the three tissues, their different effects on CAD are mainly due to their different physiological characteristics. EAT and PAT are visceral fat that provide energy to and protect the heart. Additionally, they secrete active substances under pathological conditions and become cardiotoxic, resulting in local inflammation and cardiac dysfunction.

### Limitation

First, the sample size was relatively small in our study, although we have searched Medline on the sample size of the former study to find some information of the sample size for the present design. Additionally, we have calculated the sample size and statistical power of all the factors in our study after some pre-study. Second, in our study, the blood and fat deposits tissues samples were collected during the hospitalization before the secondary prevention stragedy for CAD therapeutic treatment use. Therefore, we did not consider the impact of therapeutic treatment between CAD and the control groups. Third, the above defects of the study design can only lead to a relatively conservative conclusion, and we will try our best to go deep into some further researches on the topic.

## Conclusions

The regulation and imbalance expression of the novel biomarkers, including inflammatory adipokine, macrophage infiltration, angiogenesis, and lymphangiogenesis in EAT and PAT, may be related to the pathogenesis of CAD. The serum levels of inflammatory adipokines may correlate to CAD, which requires large sample size studies to get further validation before clinic practice.

## Supplementary Information

Supplementary Figures.

## Data Availability

All raw data will be made available on reasonable request. Requests should be directed to the corresponding author (lixiansun@126.com). Requestors will be required to sign a data access agreement to ensure the appropriate use of the study data.

## Abbreviations

CAD: Coronary artery disease
EAT: Epicardial adipose tissue
PAT: Pericardial adipose tissue
SAT: Subcutaneous adipose tissue
NCAD: Non-coronary artery disease
CTRP1: Complement-Clq TNF-related protein 1
YKL-40: Chitinase-3-like protein 1
SFRP4: Secreted frizzled-related protein 4
ADP: Adiponectin
CTRP9: Complement-Clq TNF-related protein 9
Metrnl: Meteorin-like
VEGF: Vascular endothelial growth factor
VEGFR-3: Vascular endothelial growth factor receptor 3
