# Supplementary material for: Inflammatory biomarkers, angiogenesis and lymphangiogenesis in epicardial adipose tissue correlate with coronary artery disease
Source: Sci Rep. 2023 Feb 17;13:2831. doi: 10.1038/s41598-023-30035-x (PMC9938158; doi:10.1038/s41598-023-30035-x)
Supplement: Supplementary file 1 — Supplementary Figures. [file 41598_2023_30035_MOESM1_ESM.doc]

**
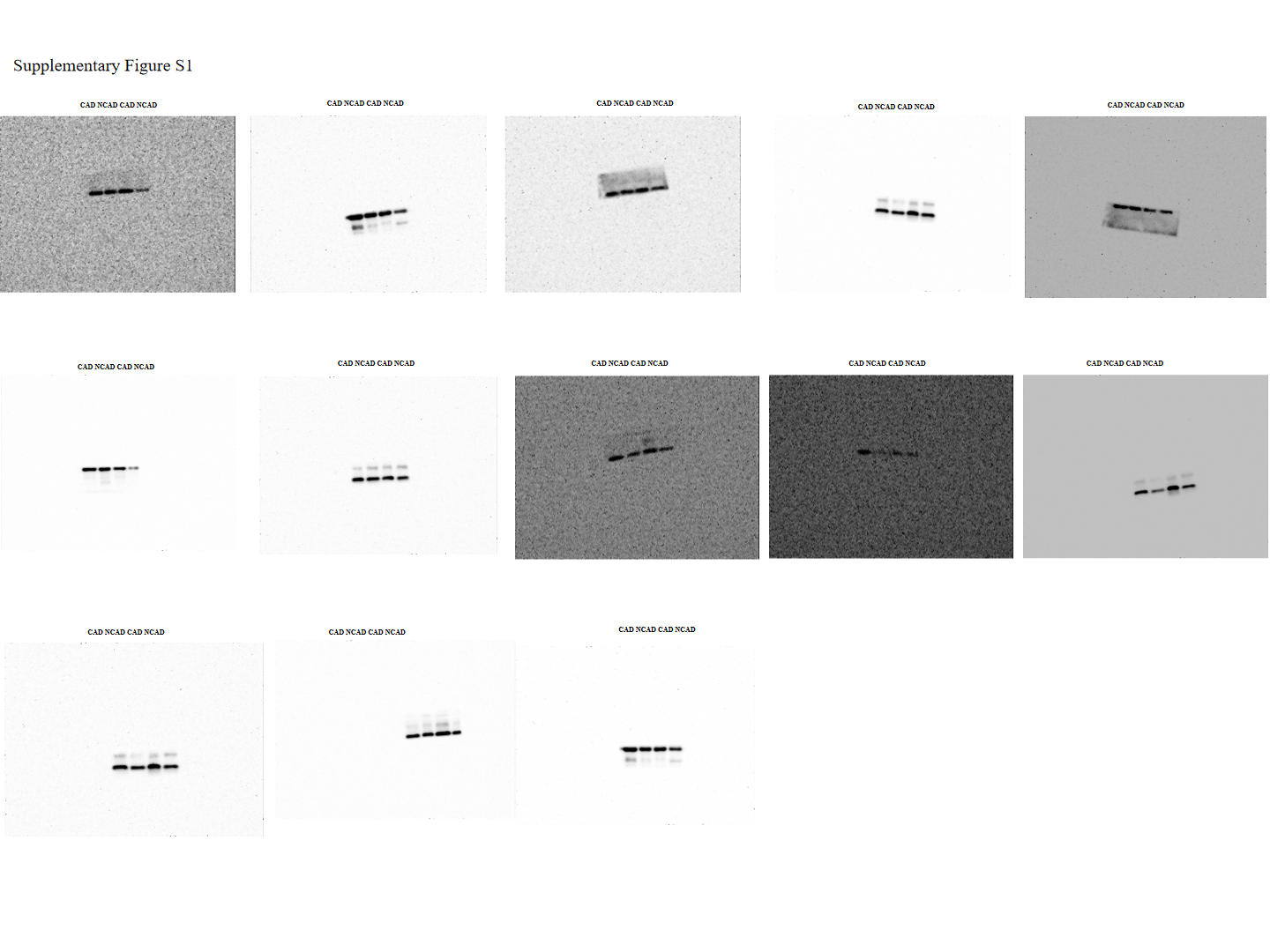
Supplementary Figure S1** Original whole CTRP1of EAT immunoblot examples with target protein bands.


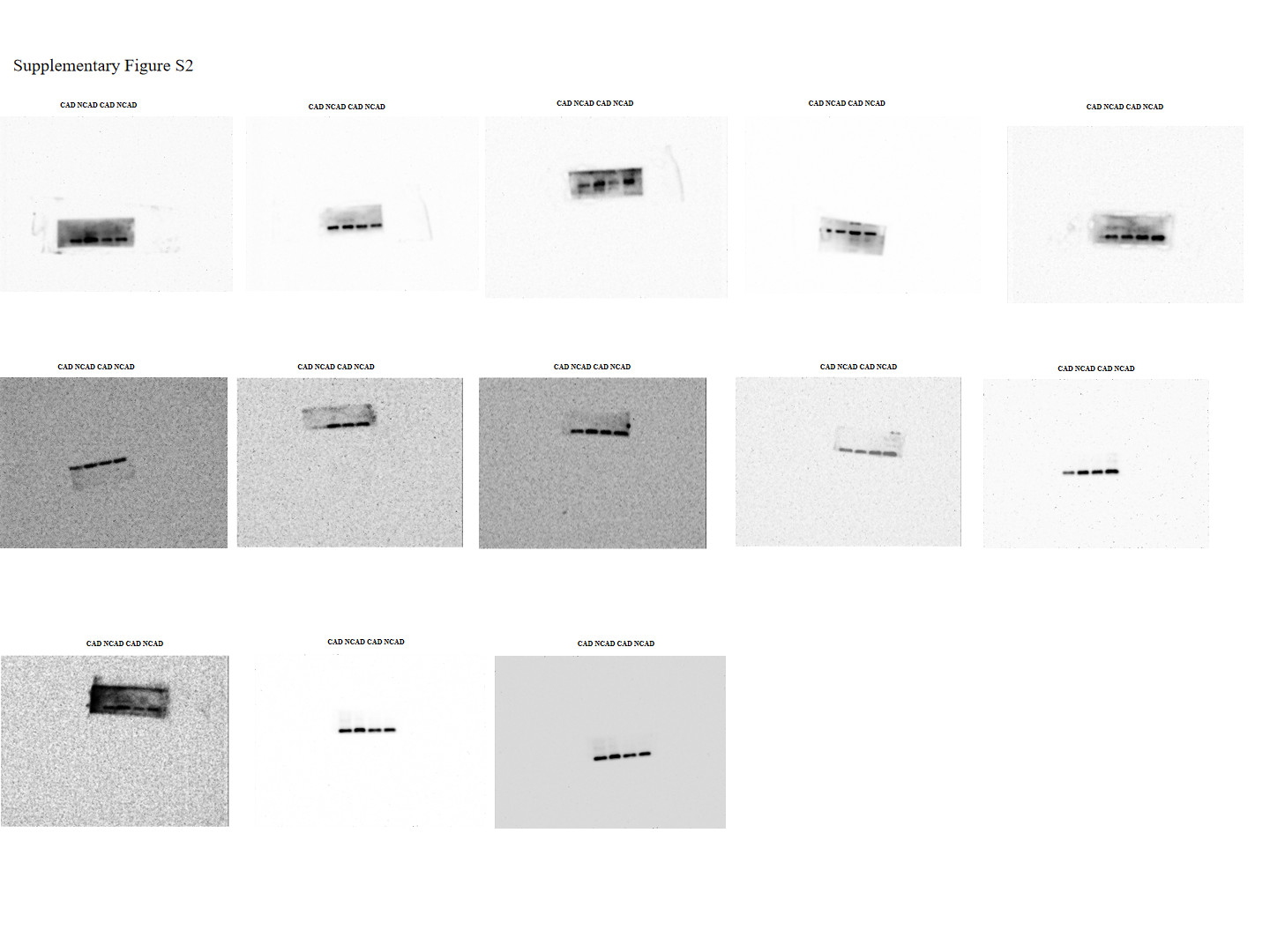


**Supplementary Figure S2** Original whole CTRP9 of EAT immunoblot examples with target protein bands.


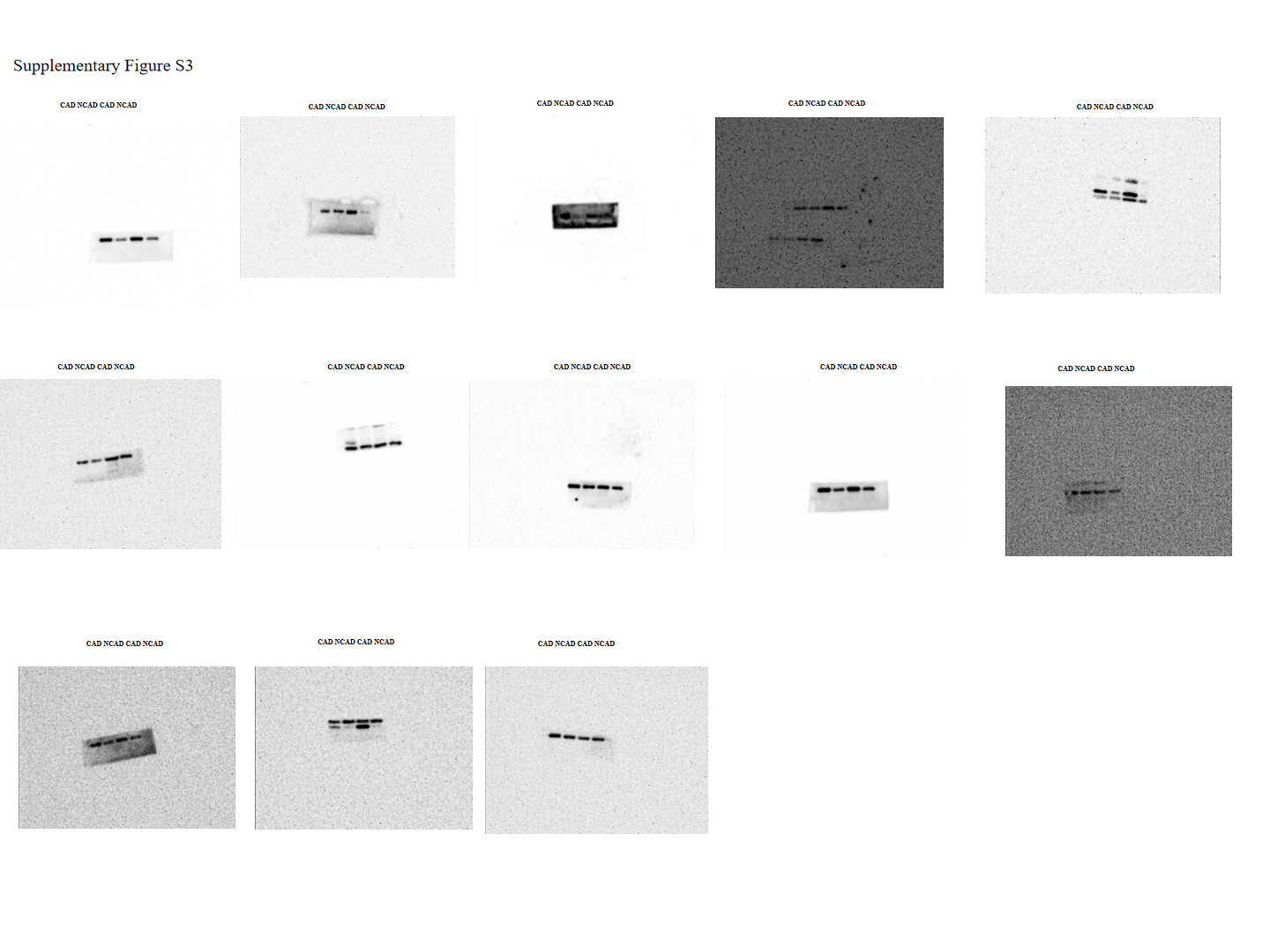


**Supplementary Figure S3** Original whole YKL-40 of EAT immunoblot examples with target protein bands.


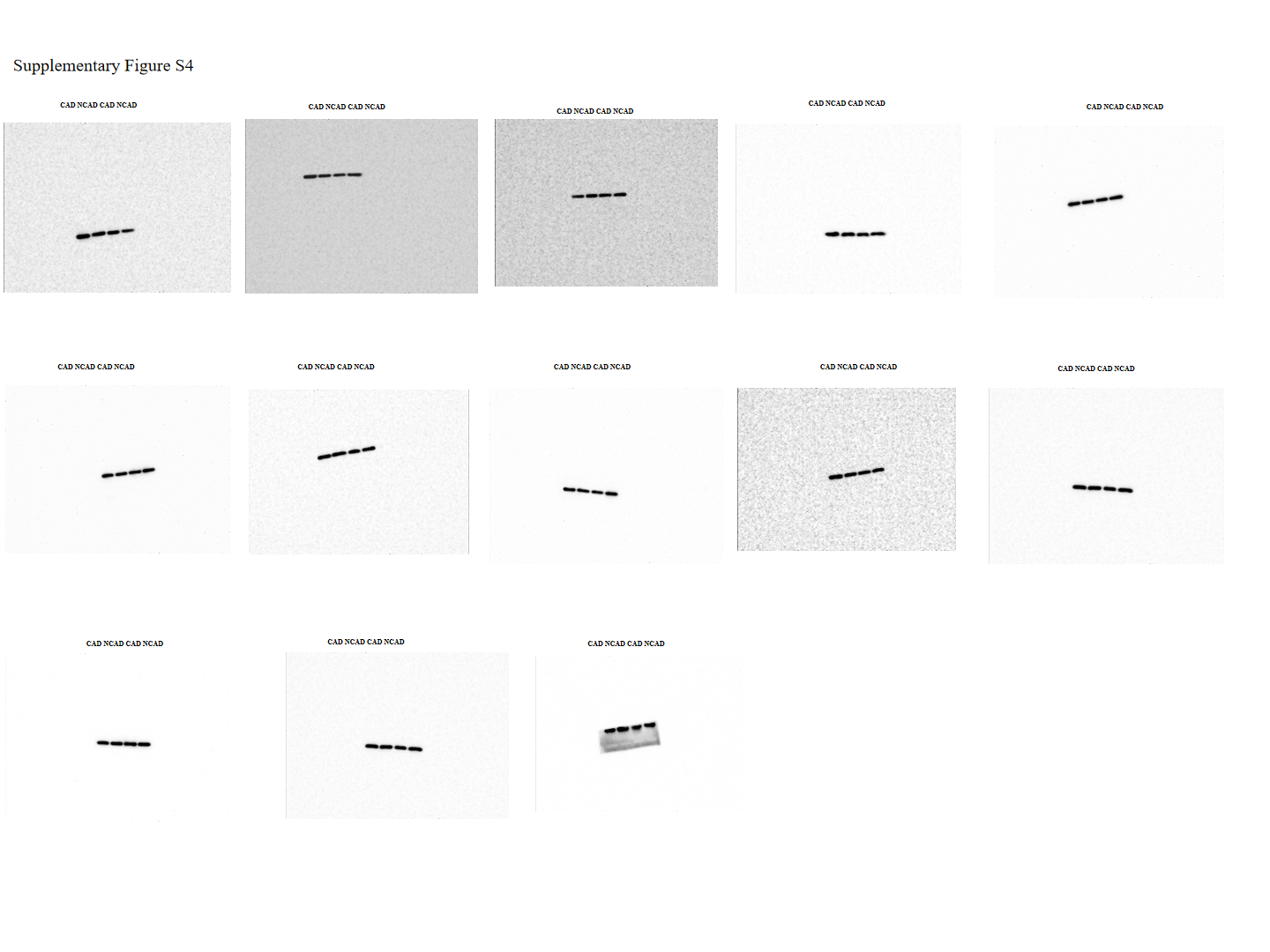


**Supplementary Figure S4** Original whole GAPDH of EAT immunoblot examples with target protein bands.


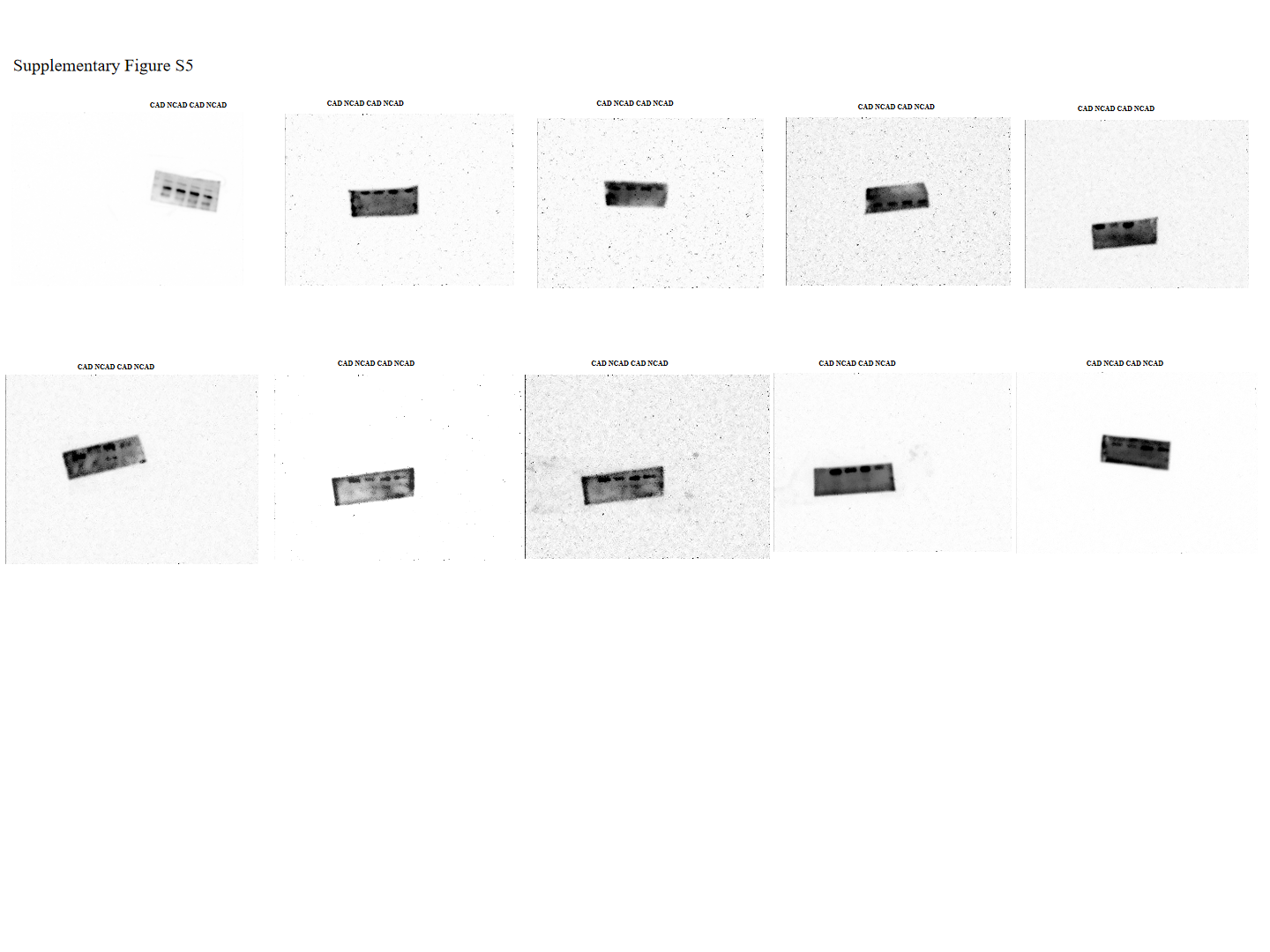


**Supplementary Figure S5** Original whole CTRP1 of PAT immunoblot examples with target protein bands.


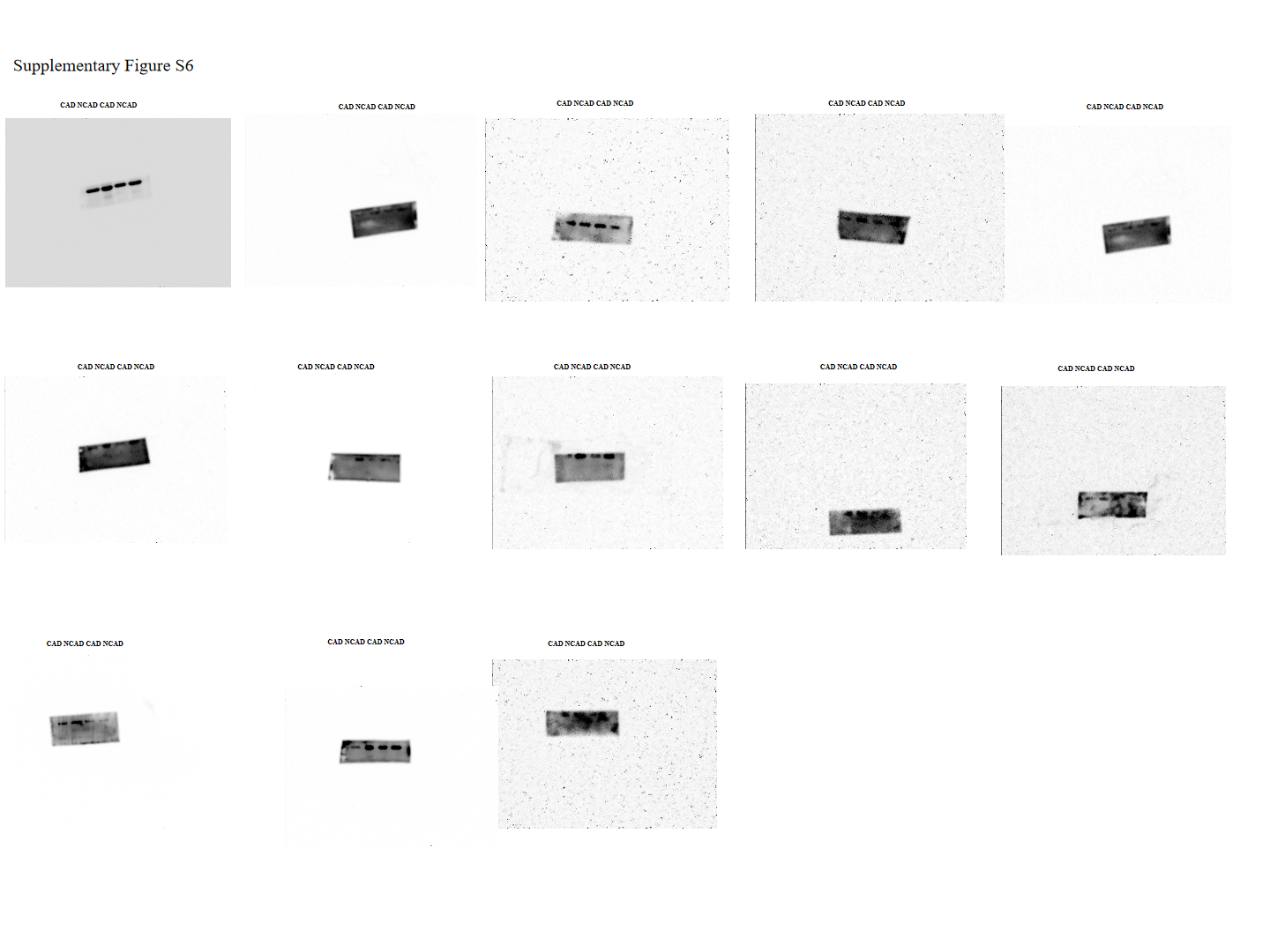


**Supplementary Figure S6** Original whole CTRP9 of PAT immunoblot examples with target protein bands.


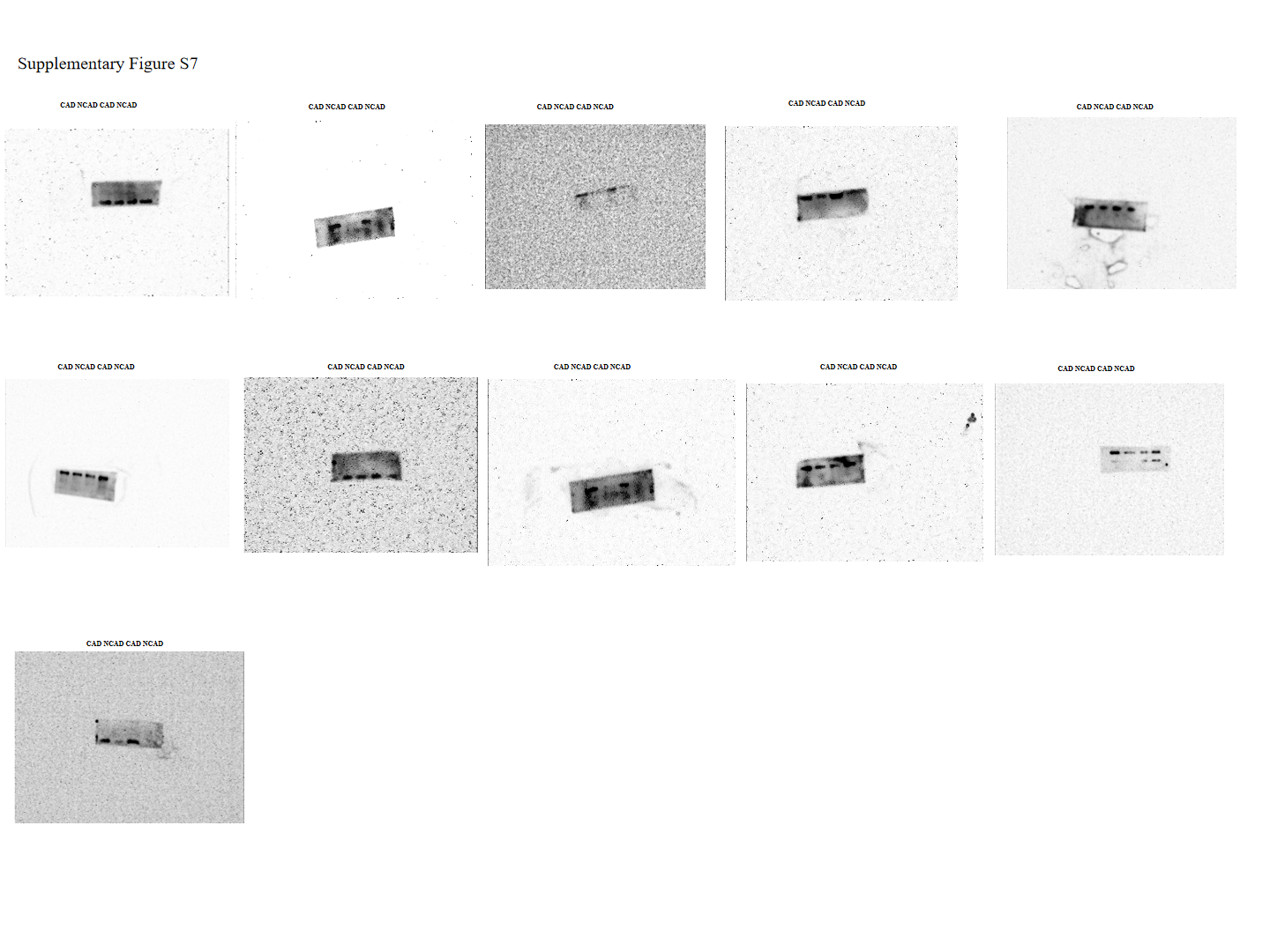


**Supplementary Figure S7** Original whole YKL-40 of PAT immunoblot examples with target protein bands.

**
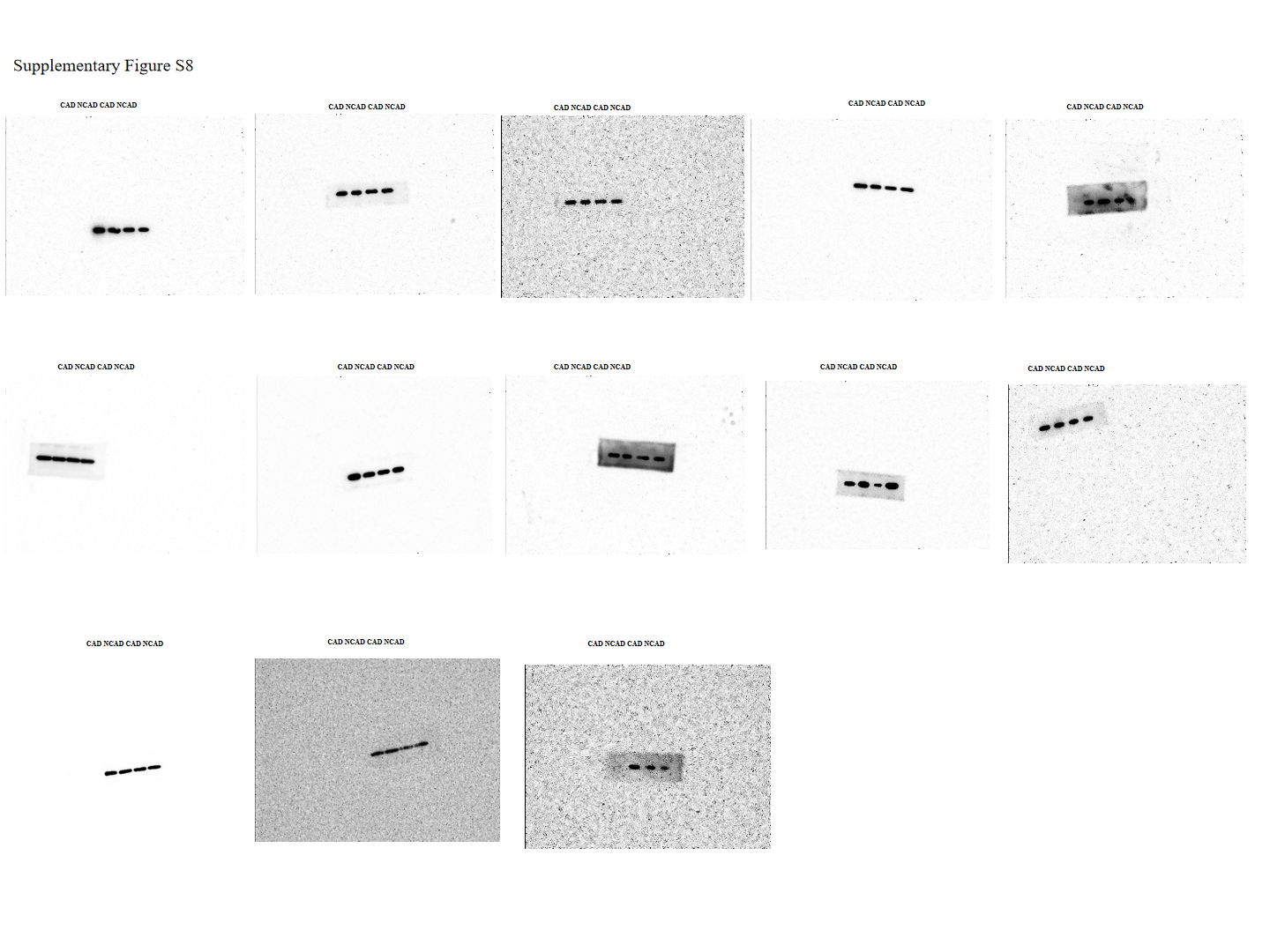
Supplementary Figure S8** Original whole GAPDH of PAT immunoblot examples with target protein bands.


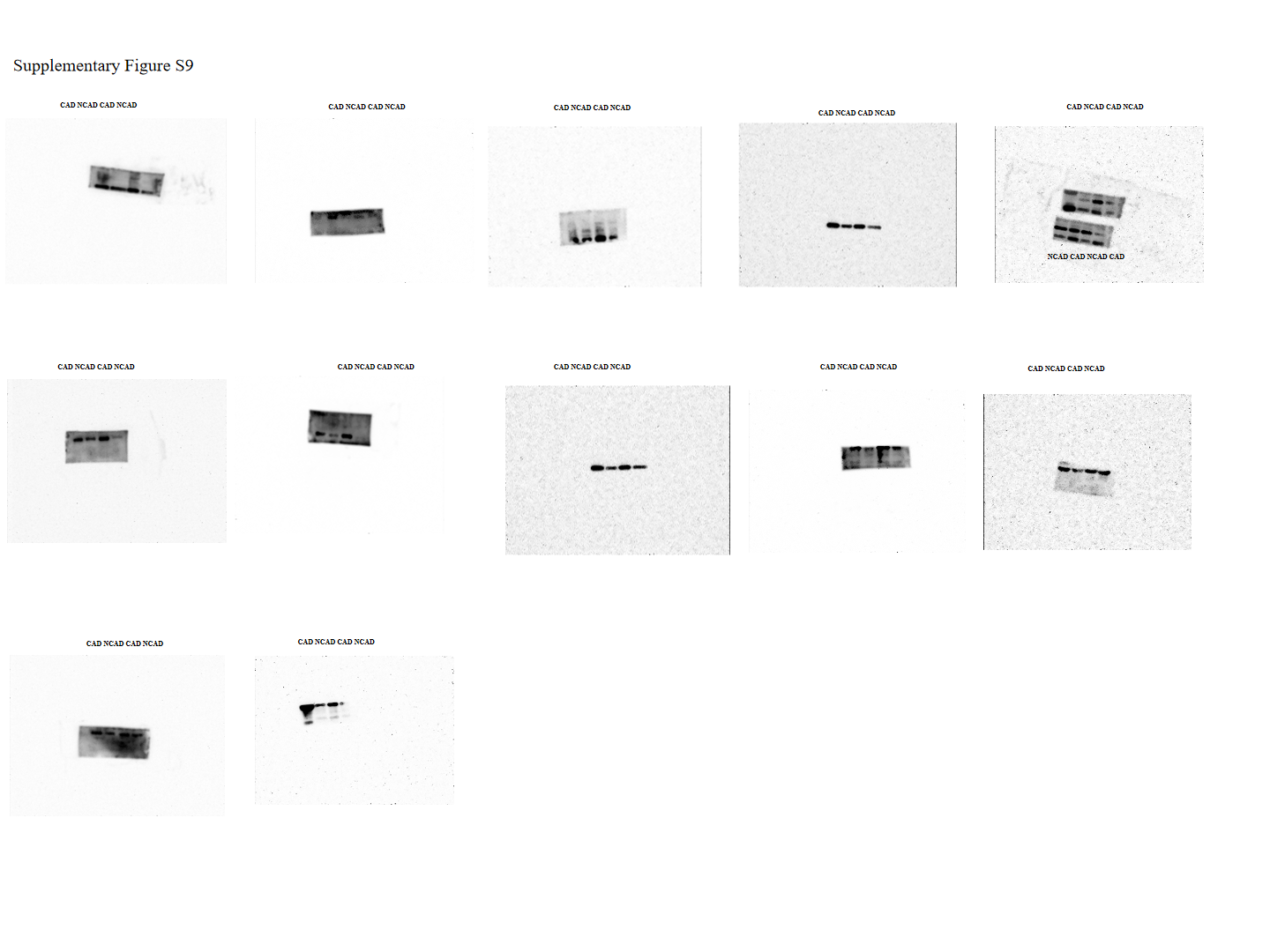


**Supplementary Figure S9** Original whole CTRP1 of SAT immunoblot examples with target protein bands.


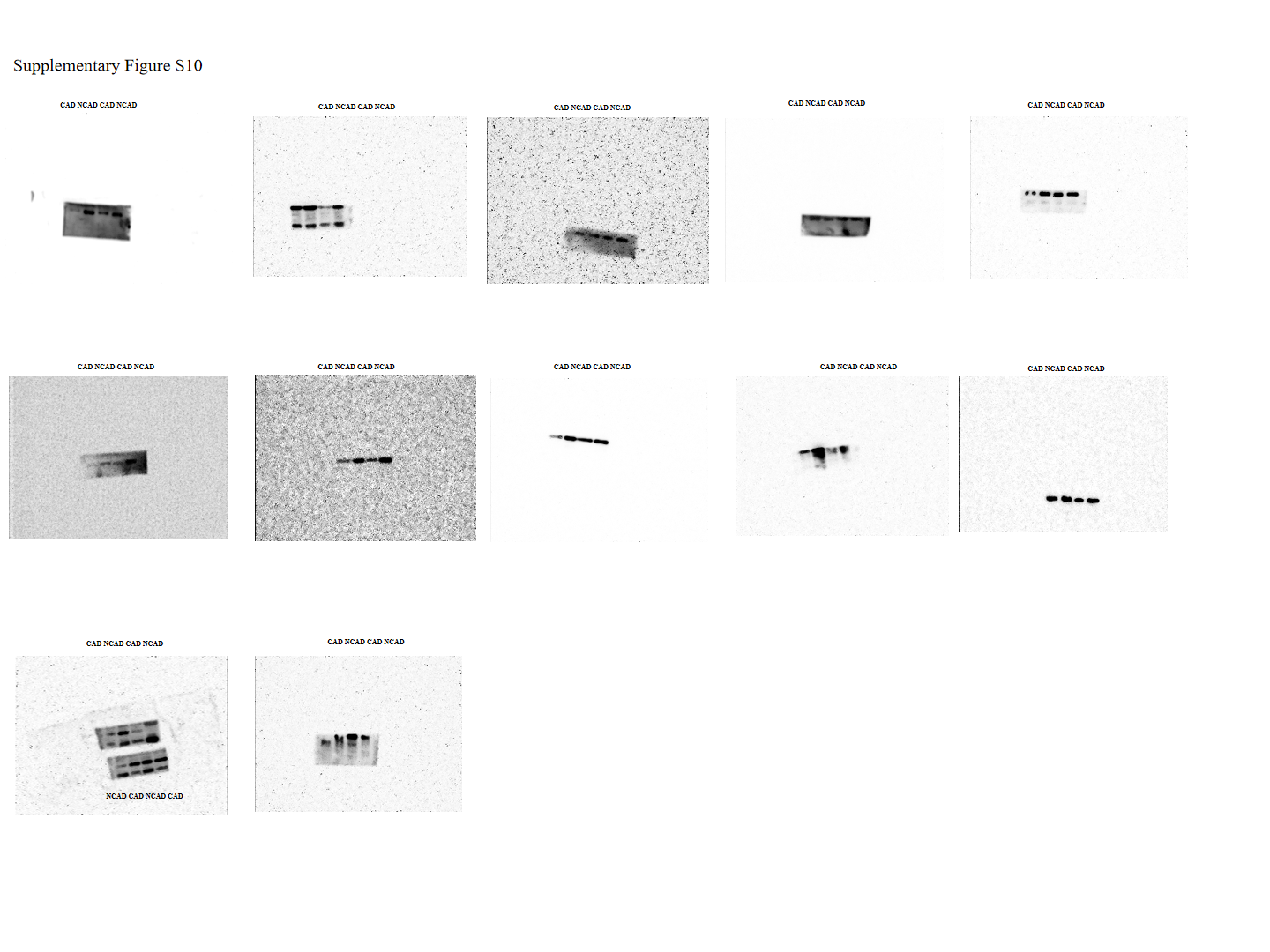


**Supplementary Figure S10** Original whole CTRP9 of SAT immunoblot examples with target protein bands.

**
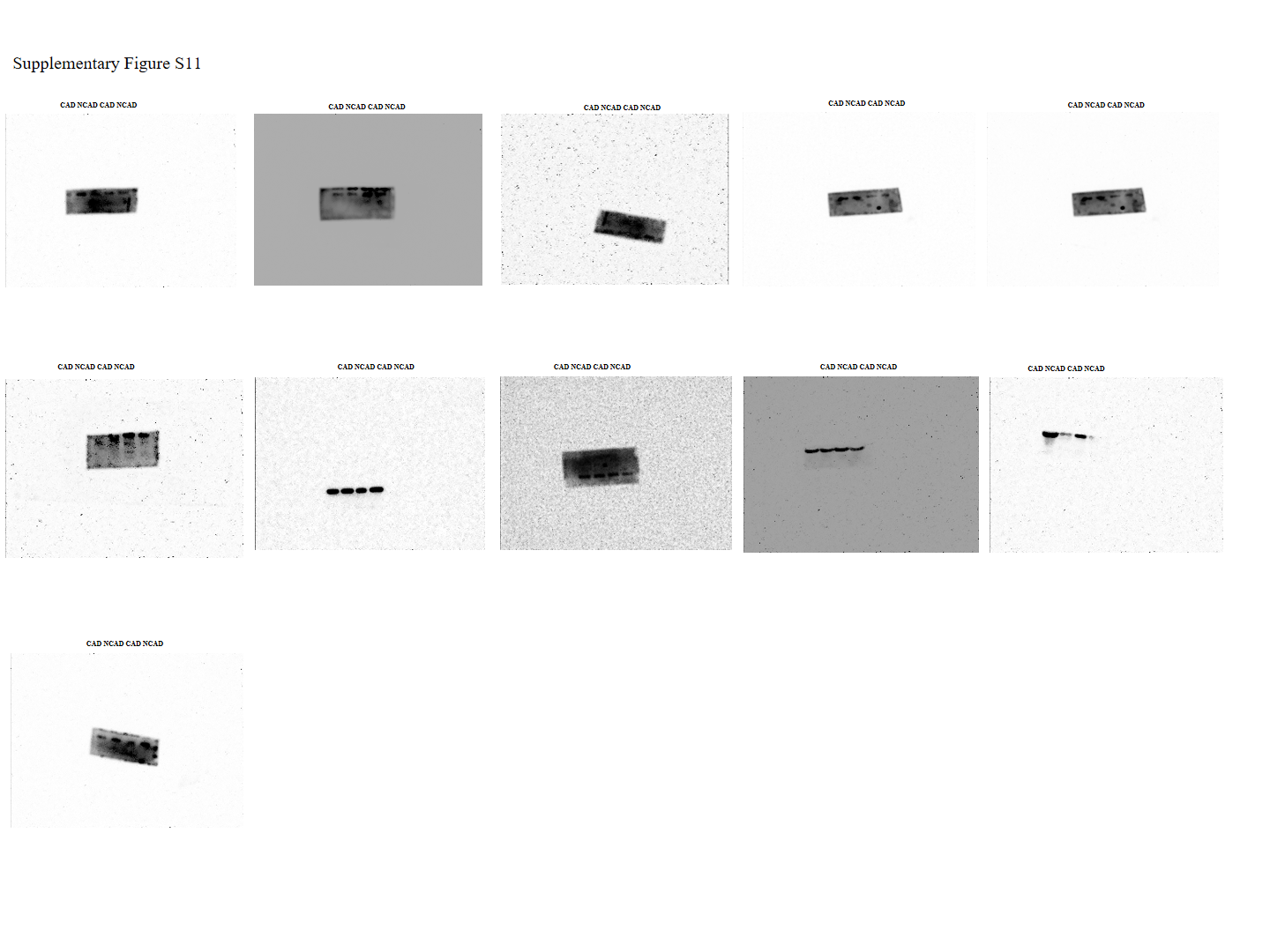
Supplementary Figure S11** Original whole YKL-40 of SAT immunoblot examples with target protein bands.


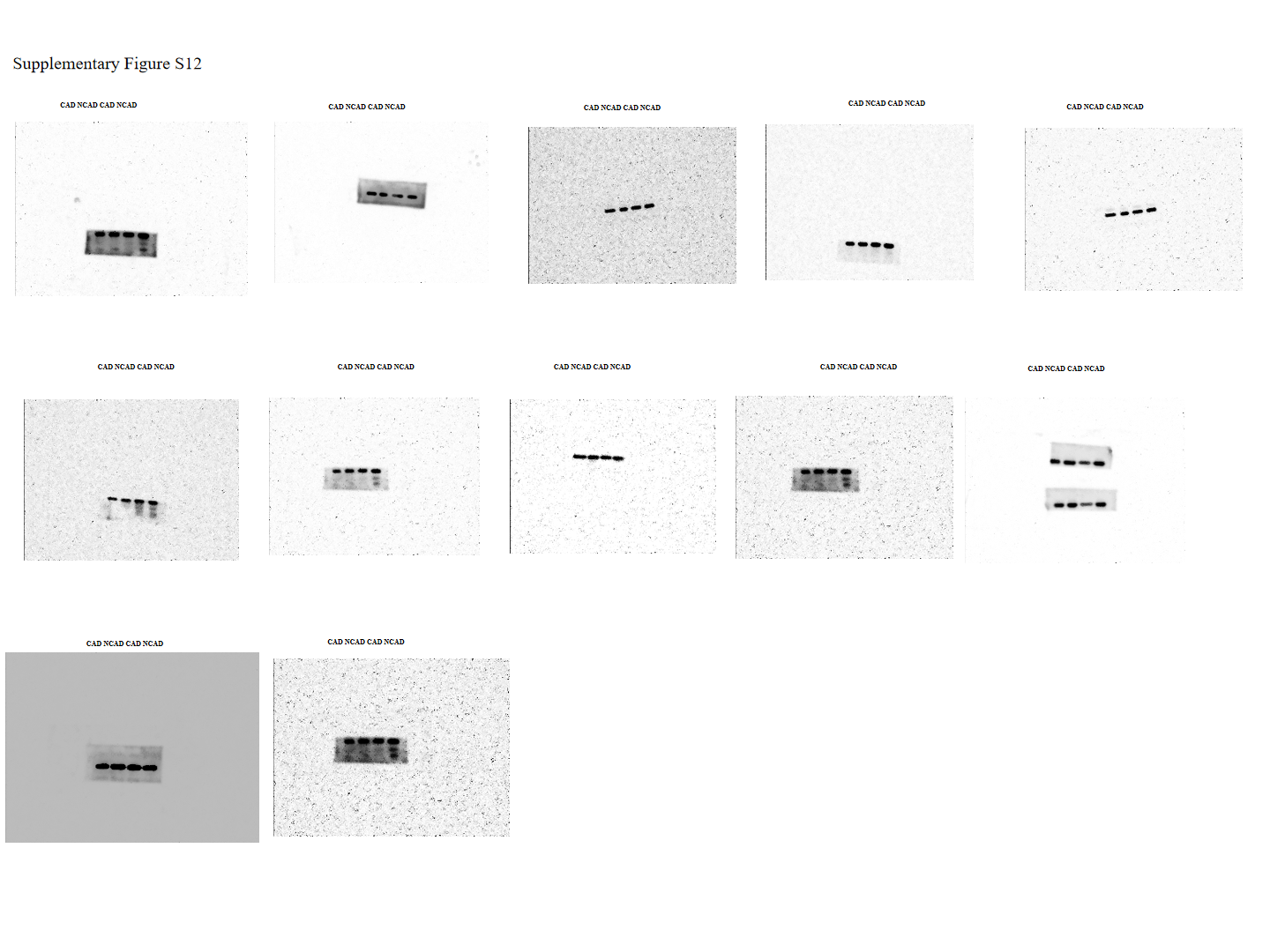


**Supplementary Figure S12** Original whole GAPDH of SAT immunoblot examples with target protein bands.
